# PubChem structure–activity relationship (SAR) clusters

**DOI:** 10.1186/s13321-015-0070-x

**Published:** 2015-07-07

**Authors:** Sunghwan Kim, Lianyi Han, Bo Yu, Volker D Hähnke, Evan E Bolton, Stephen H Bryant

**Affiliations:** National Center for Biotechnology Information, National Library of Medicine, National Institutes of Health, Department of Health and Human Services, 8600 Rockville Pike, Bethesda, MD 20894 USA

**Keywords:** PubChem, PubChem3D, Structure–activity relationship (SAR), Cluster analysis, Molecular similarity, BioSystems, MeSH

## Abstract

**Background:**

Developing structure–activity relationships (SARs) of molecules is an important approach in facilitating hit exploration in the early stage of drug discovery. Although information on millions of compounds and their bioactivities is freely available to the public, it is very challenging to infer a meaningful and novel SAR from that information.

**Results:**

Research discussed in the present paper employed a bioactivity-centered clustering approach to group 843,845 non-inactive compounds stored in PubChem according to both structural similarity and bioactivity similarity, with the aim of mining bioactivity data in PubChem for useful SAR information. The compounds were clustered in three bioactivity similarity contexts: (1) non-inactive in a given bioassay, (2) non-inactive against a given protein, and (3) non-inactive against proteins involved in a given pathway. In each context, these small molecules were clustered according to their two-dimensional (2-D) and three-dimensional (3-D) structural similarities. The resulting 18 million clusters, named “PubChem SAR clusters”, were delivered in such a way that each cluster contains a group of small molecules similar to each other in both structure and bioactivity.

**Conclusions:**

The PubChem SAR clusters, pre-computed using publicly available bioactivity information, make it possible to quickly navigate and narrow down the compounds of interest. Each SAR cluster can be a useful resource in developing a meaningful SAR or enable one to design or expand compound libraries from the cluster. It can also help to predict the potential therapeutic effects and pharmacological actions of less-known compounds from those of well-known compounds (i.e., drugs) in the same cluster.

**Graphical abstract:**

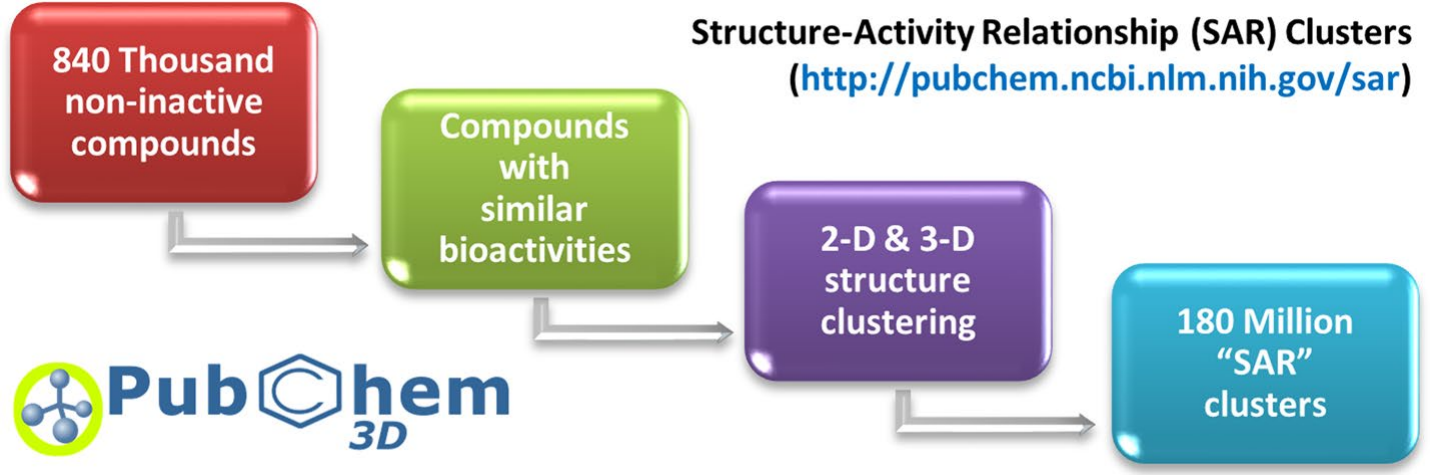

**Electronic supplementary material:**

The online version of this article (doi:10.1186/s13321-015-0070-x) contains supplementary material, which is available to authorized users.

## Background

PubChem [1–6] is a public repository for information on small molecules and their biological activities (hereafter simply called “bioactivities”). It has a wealthy collection of chemical information, with more than 180 million depositor-provided substance descriptions, 60 million unique chemical structures, and one million biological assay results (as of December 2014). These biological assays cover more than 8,000 unique protein target sequences. PubChem’s bioactivity data contents include those from the U.S. National Institutes of Health (NIH)’s Molecular Libraries Program [7], manually extracted results pulled from tens of thousands of scientific papers published in medicinal chemistry journals by data contributors such as ChEMBL [8], and beyond.

For the efficient use of this vast amount of chemical information, PubChem provides various search and analysis tools, most of which exploit the concept of molecular similarity. PubChem can quickly quantify similarity between chemical structures at a rate of millions of pairwise comparisons per CPU core per second, using a fragment-based two-dimensional (2-D) similarity method that employs the 881-bit PubChem subgraph fingerprints [9] and the Tanimoto equation [10–12] (see the “Methods” section for more details). However, traditional 2-D similarity methods sometimes fail to recognize structural similarity that can be easily realized with three-dimensional (3-D) similarity methods [13–16]. To address this issue, the PubChem3D project was launched [17–24]. PubChem3D generates 3-D conformer models for about 92% of chemical records in PubChem, averaging ~110 conformers per compound [17, 24]. It also delivers tools and services that exploit 3-D molecular similarity between these conformer models, which is quantified using the atom-centered Gaussian-shape comparison method by Grant and Pickup [25–28] (see the “Methods” section for more details on PubChem’s 3-D similarity method). To understand the statistical meaning of PubChem 2-D and 3-D similarity scores, the similarity score distributions for randomly selected biologically tested compounds were investigated using both a single conformer [22] and multiple conformers [23] for each compound. In addition, PubChem3D pre-computes compounds similar to each applicable compound in PubChem in terms of 3-D similarity, and provides immediate access to these “3-D neighbors” as well as their respective superpositions [19]. Our previous studies demonstrate the utility of the PubChem3D resources by illustrating complementarity between PubChem 2-D and 3-D similarity methods [19, 21–23]. The present study describes our preliminary work to build a new database resource from the PubChem3D project, namely, PubChem structure–activity relationship (SAR) clusters [29].

Currently, two million compounds in PubChem have been tested in at least one assay, with 48% of them (0.96 million compounds) declared active in at least one assay. Extracting valuable SARs from such a large corpus of bioactivity information may provide new opportunities for facilitating drug discovery and development. However, it is not an easy task because of the heterogeneous nature of these data. Because biological assays in PubChem are contributed by many data depositors, these assays reflect different interests of the individual depositors. Therefore, biological assays that target the same protein or pathway may test different sets of compounds (typically with different scaffolds). Even if these assays do test some common compounds, the experimental conditions used in the assays are not necessarily identical, making it difficult to compare bioactivity data from different assays. In addition, the majority of these data were generated from high throughput screenings, which are known to contain many false positives/negatives [30–32]. Despite these difficulties, there has been an increasing interest in systematic large-scale mining of SARs from bioactivity data available in the public domain [33, 34].

The present study employed a bioactivity-centric clustering approach to group more than 800 thousand “non-inactive” compounds archived in PubChem according to their structural similarity and bioactivity similarity. In this study, a non-inactive compound is defined as any molecule that is *not* declared to be inactive in a biological assay. This includes “unspecified/inconclusive” compounds as well as “active” molecules. The reason for using non-inactive compounds instead of active compounds is that the unspecified and inconclusive compounds are indeed active in many assays. (See the “Methods” section for more details on the definition of non-inactive compounds.) Clustering these non-inactive compounds resulted in 18 million SAR clusters, each of which contains a group of structurally similar molecules that have similar bioactivities. Importantly, three different contexts of bioactivity similarity were considered. Compounds can have similar bioactivities to each other when they were tested to be non-inactive: (1) in a common assay, (2) against a common protein sequence, or (3) against proteins involved in a common biological pathway. The use of the three contexts of bioactivity similarity allows for organizing bioactivity data of molecules tested in a single assay, as well as those scattered across multiple assays that are targeting the same protein or pathway. In addition, five different structural similarity measures (one 2-D and four 3-D similarity measures) were used to reflect different flavors of chemical structure similarity that may be unrecognizable when only one measure is employed. As a result, each of the SAR clusters belongs to one of fifteen different cluster types (arising from combination of each of the three bioactivity similarity contexts with each of the five different structural similarity measures: 3 contexts × 5 measures = 15 cluster types). The detailed procedures for generating the SAR clusters are described in the present paper, with discussion on effects of the 2-D and 3-D similarity measures upon the clustering results.

## Results

### Construction of three data sets

To consider three different contexts of bioactivity similarity between molecules, three different compound sets (**Sets A**, **B**, and **C**) were constructed with PubChem Compound records that had 3-D information available *and* that satisfied the following conditions:

1. for **Set A**, compounds were declared to be non-inactive in at least one bioassay stored in the PubChem BioAssay database [3–5] (unique identifier: AID),
2. for **Set B**, compounds were declared to be non-inactive against at least one target protein sequence that was archived in the NCBI’s Protein database [6] (unique identifier: GI), and
3. for **Set C**, compounds were declared to be non-inactive against at least one target protein sequence involved in a biological pathway or biosystem that was stored in the NCBI’s BioSystems database [35] (unique identifier: BSID).

More detailed descriptions on construction of these sets, including the definition of the non-inactive compounds, are given in the “Methods” section. Although any database can have unique identifiers (UIDs) to organize its records, the term “UID” is specifically reserved in the present study for any of AID, GI, and BSID (depending on the context) to represent the three contexts of bioactivity similarity, but not for CID (the unique identifier used in the PubChem Compound database). Note that a single protein sequence may have multiple GIs in the Protein database. As explained in detail in the “Methods” section, this issue was addressed by using the protein identity group (PIG), which disambiguates different GIs that have an identical protein sequence. The use of the PIG allowed for treating identical protein sequences as one record and removing redundancy in the protein sequences considered in the present study. A side effect of this is that it groups identical protein sequences from different organisms.

As listed in Table 1, **Set A** had 843,845 compounds associated with 548,071 assays, **Set B** had 400,599 compounds associated with 4,280 unique GIs, and **Set C** had 265,470 compounds associated with 4,540 BSIDs. Note that not all biological assays archived in PubChem have information on target proteins, and that not all target proteins have associated pathways in the BioSystems database. [That is, **Set A** includes **Set B**, which in turn includes **Set C**.] As a result, **Set A** has the largest number of compounds and **Set C** has the smallest.

**Table 1:** Counts of compounds (CIDs), assays (AIDs), proteins (GIs), and pathways (BSIDs) with PubChem structure–activity relationship (SAR) clustering results as a function of similarity type

	Initial	3-D clusters				2-D clusters	Any clusters
		*ST* ^*ST*-*opt*^	*ComboT* ^*ST*-*opt*^	*CT* ^*CT*-*opt*^	*ComboT* ^*CT*-*opt*^		
Number of CIDs							
Assay-centric clusters (from **Set A**)	843,845	669,504 (79.3%)	746,042 (88.4%)	747,969 (88.6%)	747,586 (88.6%)	802,383 (95.1%)	829,279 (98.3%)
Protein-centric clusters (from **Set B**)	400,599	313,282 (78.2%)	356,954 (89.1%)	360,200 (89.9%)	357,543 (89.3%)	382,737 (95.5%)	397,197 (99.2%)
Pathway-centric clusters (from **Set C**)	265,470	213,738 (80.5%)	243,006 (91.5%)	245,215 (92.4%)	243,378 (91.7%)	257,170 (96.9%)	264,338 (99.6%)
Number of UIDs							
Assay-centric clusters (from **Set A**)	548,071	218,789 (39.9%)	244,381 (44.6%)	245,334 (44.8%)	246,625 (45.0%)	264,311 (48.2%)	274,435 (50.1%)
Protein-centric clusters (from **Set B**)	4,280	3,340 (78.0%)	3,419 (79.9%)	3,438 (80.3%)	3,428 (80.1%)	3,620 (84.6%)	3,660 (85.5%)
Pathway-centric clusters (from **Set C**)	4,540	3,973 (87.5%)	4,073 (89.7%)	4,097 (90.2%)	4,089 (90.1%)	4,149 (91.4%)	4,168 (91.8%)

Numbers in parentheses are percentages of the counts relative to the total count initially considered for each cluster set type. The UID represents AID, GI, and BSID for assay-, protein-, and pathway-centric clusters, respectively.

### Construction of SAR clusters

To generate SAR clusters for each of the UIDs (i.e., 548,071 AIDs, 4,280 GIs, and 4,540 BSIDs), the non-inactive compounds associated with that UID were retrieved from the appropriate data set (i.e., **Set A** for AIDs, **Set B** for GIs, **Set C** for BSIDs) and grouped by structural similarity, using the Taylor–Butina grouping algorithm [36, 37], as implemented in the software provided by Mesa Analytics and Computing, Inc. [38, 39]. The structural similarity between compounds was quantified with five similarity measures ($ST^{{ST{\text -}opt}}$, $ComboT^{{ST{\text -}opt}}$, $CT^{{CT{\text -}opt}}$, $ComboT^{{CT{\text -}opt}}$, and the 2-D Tanimoto), as defined in the “Methods” section, and the clustering thresholds (*d*^*thresh*^ in Table 2) were derived from the summary statistics of these similarity measures [23]. A more detailed description for PubChem SAR clustering is given in the “Methods” section.

**Table 2:** Average ($\bar{x}$) and standard deviation (*s*) of the similarity scores between 10,000 randomly-selected biologically-tested compounds (from Ref. [22, 23]), and the dissimilarity threshold (*d* ^*thresh*^) used in the present study to generate the structure–activity relationship (SAR) clusters

Similarity measures	$\bar{x}$	*s*	$\bar{x}$ + 2*s*	*d* ^*thresh*^
2-D	0.4229	0.1326	0.6881	0.3119
3-D (*N*_*max*_ = 1)				
*ST* ^*ST*-*opt*^	0.5438	0.0986	0.7410	–
*ComboT* ^*ST*-*opt*^	0.6161	0.1276	0.8713	–
*CT* ^*CT*-*opt*^	0.1807	0.0609	0.3024	–
*ComboT* ^*CT*-*opt*^	0.5859	0.1440	0.8738	–
3-D (*N*_*max*_ = 10)				
*ST* ^*ST*-*opt*^	0.6464	0.1017	0.8498	0.1502
*ComboT* ^*ST*-*opt*^	0.7682	0.1337	1.0356	0.4822
*CT* ^*CT*-*opt*^	0.2485	0.0706	0.3898	0.6102
*ComboT* ^*CT*-*opt*^	0.7733	0.1386	1.0505	0.4748

*N*_*max*_ is the maximum number of diverse conformers considered per compound for the 3-D similarity computation. The *d* ^*thresh*^ value for each of the five similarity measures were determined by subtracting its ($\bar{x}$ + 2*s*) value from unity (after normalization to one for *ComboT* ^*ST*-*opt*^ and *ComboT* ^*CT*-*opt*^). The statistical parameters for *N*_*max*_ = 10 were used to determine the *d* ^*thresh*^ value for the 3-D similarity measures.

The resulting clusters can be broadly classified into three types, according to the context of the bioactivity similarity considered. An “assay-centric” SAR cluster (generated from **Set A**) is defined as a group of structurally similar compounds tested to be non-inactive in a common bioassay. A “protein-centric” SAR cluster (generated from **Set B**) is defined as a group of structurally similar compounds declared to be non-inactive against a common protein target, and a “pathway-centric” SAR cluster (generated from **Set C**) is defined as a group of structurally similar compounds declared non-inactive against protein targets involved in a common biological pathway. Alternatively, the clusters can be categorized into five types according to the structural similarity measures employed: $ST^{{ST{\text -}opt}}$, $ComboT^{{ST{\text -}opt}}$, $CT^{{CT{\text -}opt}}$, $ComboT^{{CT{\text -}opt}}$, and 2-D clusters. Note that all of the first four clusters are 3-D clusters. Combination of the three bioactivity similarity contexts and the five structural similarity measures leads to 15 SAR cluster subtypes.

### Summary statistics of SAR clusters

**Figure 1:**
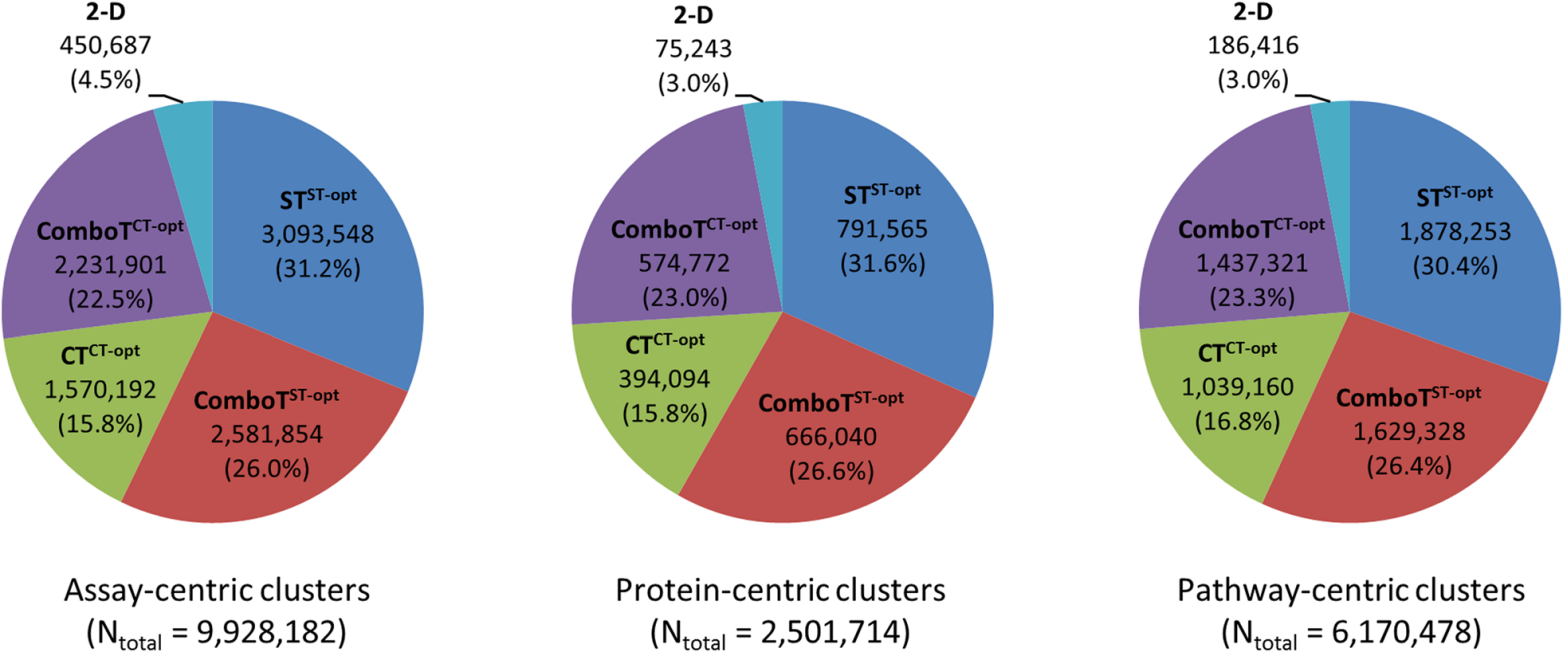
Number of structure–activity relationship (SAR) clusters. These numbers do not include clusters with only one compound (i.e., singletons). 3-D clusters that have multiple conformers of only one compound were also regarded as singletons and not included in the statistics. *N*_*total*_ indicates the total number of clusters for a given bioactivity similarity context. *Numbers in parentheses* on the pie charts indicate the percentage of five cluster types (based on structural similarity measures used in clustering) with respect to *N*_*total*_ for the corresponding bioactivity similarity context. For all three bioactivity similarity contexts, there are more 3-D clusters than 2-D clusters.

The numbers of SAR clusters generated for the 15 cluster subtypes are compared in Figure 1. There were 9.9 million assay-centric clusters, 2.5 million protein-centric clusters, and 6.2 million pathway-centric clusters. If the five similarity measures employed give similar clustering results, the number of SAR clusters from a given similarity measure is expected to be around 20% of the total number of clusters. However, for all three bioactivity similarity contexts, 2-D clusters corresponded only to 3–4.5% of the total clusters. All remaining clusters were 3-D clusters.

**Figure 2:**
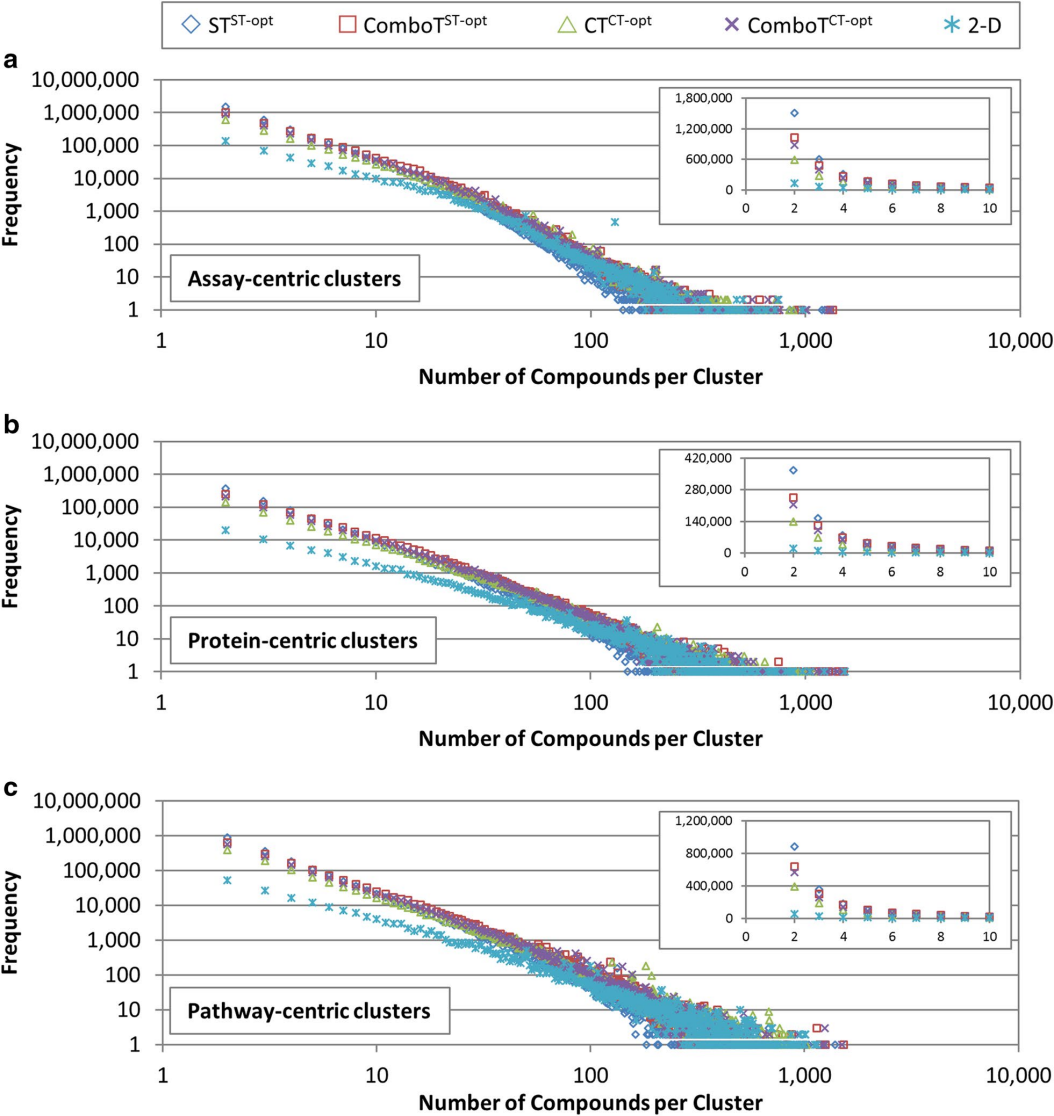
Distribution of 2-D and 3-D cluster sizes in terms of the number of “compounds” per cluster.* Panels* **a**, **b** and **c** are for assay-, protein-, and pathway-centric clusters, respectively. The proportion of small clusters (e.g., with two or three compounds) are much greater for 3-D clusters than for 2-D clusters. This may be related to the use of multiple conformers per compound for 3-D clustering.

The summary statistics for the SAR clusters are shown in Table 3. The average size of the 2-D clusters was greater than that of 3-D clusters. For example, for the assay-centric clusters, the 2-D clusters had 8.2 compounds on average, but the 3-D clusters contained 4.0–5.9 compounds on average, depending on the 3-D similarity measure employed. This trend is well reflected in Figure 2, which shows the distributions of the cluster sizes in terms of the number of compounds per cluster. Each cluster had at least two *compounds* because all singletons were removed. When a 3-D cluster contained multiple conformers of only one compound and nothing else, the cluster was considered as a singleton.

**Table 3:** Summary statistics of structure–activity relationship (SAR) clusters

	3-D clusters								2-D clusters	
	*ST* ^*ST*-*opt*^		*ComboT* ^*ST*-*opt*^		*CT* ^*CT*-*opt*^		*ComboT* ^*CT*-*opt*^			
	$\bar{x}$	*s*	$\bar{x}$	*s*	$\bar{x}$	*s*	$\bar{x}$	*s*	$\bar{x}$	*s*
Assay-centric clusters										
# Compounds per cluster	4.0	5.2	5.3	7.8	5.9	9.5	5.4	8.3	8.2	13.8
# Conformers per cluster	5.8	11.5	10.3	25.4	18.3	48.2	12.2	32.0	–	–
# Clusters per compound	18.6	67.7	18.3	80.4	12.4	51.1	16.2	70.8	4.6	18.8
# Clusters per UID	14.1	55.8	10.6	48.9	6.4	29.3	9.1	42.3	1.7	6.3
Target-centric clusters										
# Compounds per cluster	4.7	9.0	6.7	14.5	7.9	19.2	6.9	15.8	13.7	33.8
# Conformers per cluster	6.3	18.4	11.4	40.8	21.4	84.9	13.6	52.8	–	–
# Clusters per compound	11.8	39.9	12.4	47.0	8.7	31.0	11.1	41.3	2.7	8.9
# Clusters per UID	237.0	463.0	194.8	389.9	114.6	232.0	167.7	340.6	20.8	40.5
Pathway-centric clusters										
# Compounds per cluster	4.7	8.7	6.5	13.7	7.4	18.1	6.6	14.8	13.5	35.1
# Conformers per cluster	6.4	17.9	11.1	37.2	19.4	79.1	12.9	47.4	–	–
# Clusters per compound	41.5	119.9	43.2	121.1	31.3	93.1	39.1	110.8	9.8	26.3
# Clusters per UID	472.8	774.1	400.0	683.9	253.6	439.7	351.5	607.2	44.9	70.2

Symbols $\bar{x}$ and *s* indicate the average and standard deviation, respectively. UID represents AID, GI, and BSID for assay-, protein-, and pathway-centric clusters, respectively. Statistics exclude singleton clusters.

**Figure 3:**
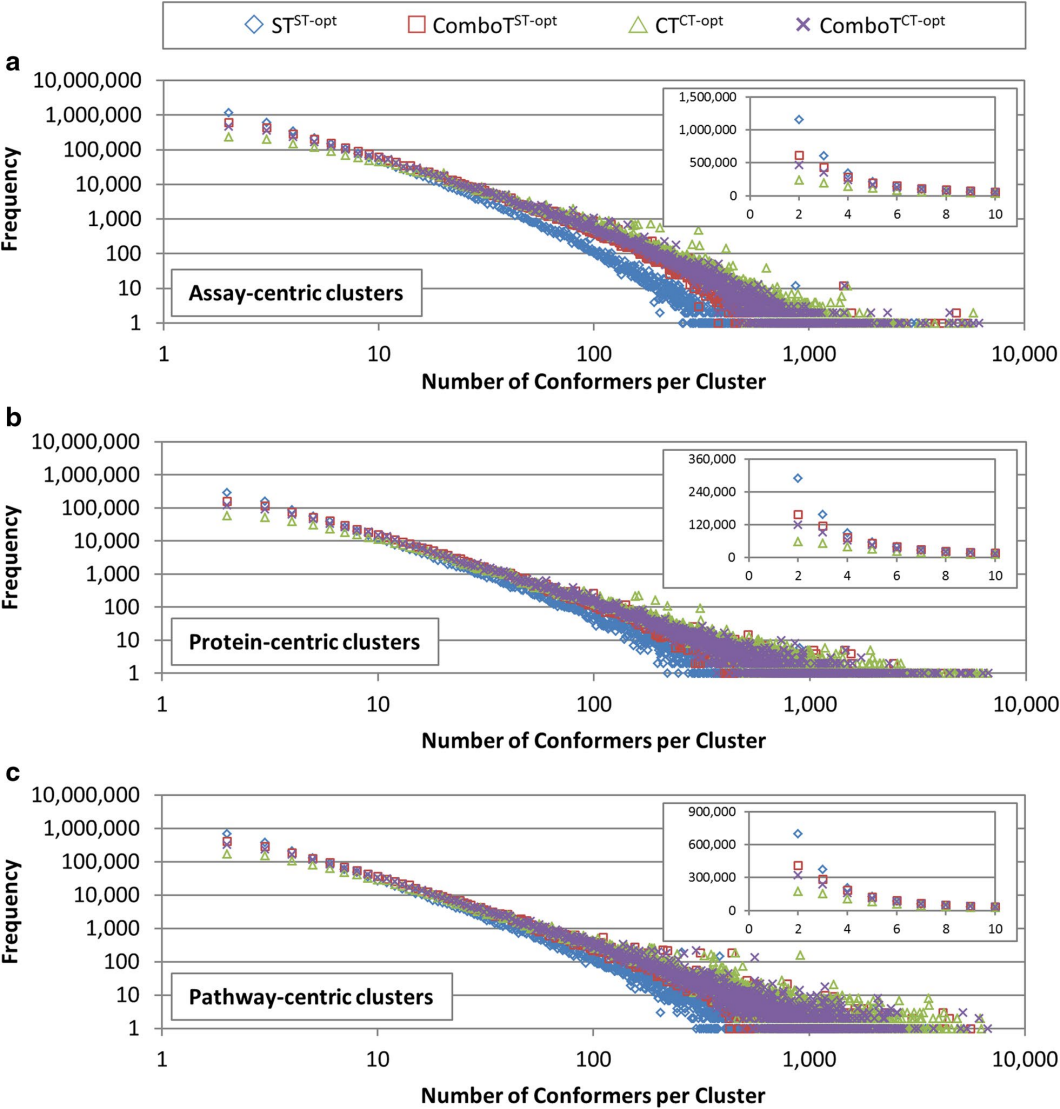
Distribution of 3-D cluster sizes in terms of the number of “conformers” per cluster.* Panels* **a**, **b** and **c** are for assay-, protein-, and pathway-centric clusters, respectively. Data for 2-D clusters are not shown because 2-D clustering does not use conformers.

The distribution of the cluster sizes in terms of the number of conformers per cluster is displayed in Figure 3. Only the 3-D cluster data are shown because the 2-D clustering does not use conformer models. Figure 3 clearly shows that, for all three bioactivity similarity contexts, the proportion of small clusters (e.g., with two or three conformers) increases in order of $CT^{{CT{\text -}opt}}$ < $ComboT^{{CT{\text -}opt}}$ < $ComboT^{{ST{\text -}opt}}$ < $ST^{{ST{\text -}opt}}$ clusters. This trend is reflected in the average number of conformers per cluster (listed in Table 3), which increases in order of $ST^{{ST{\text -}opt}}$ < $ComboT^{{ST{\text -}opt}}$ < $ComboT^{{CT{\text -}opt}}$ < $CT^{{CT{\text -}opt}}$ clusters. This order in the cluster size among the four 3-D cluster types remains unchanged when the number of compounds per cluster is used as a measure of the cluster size (as shown in Figure 2; Table 3).

**Figure 4:**
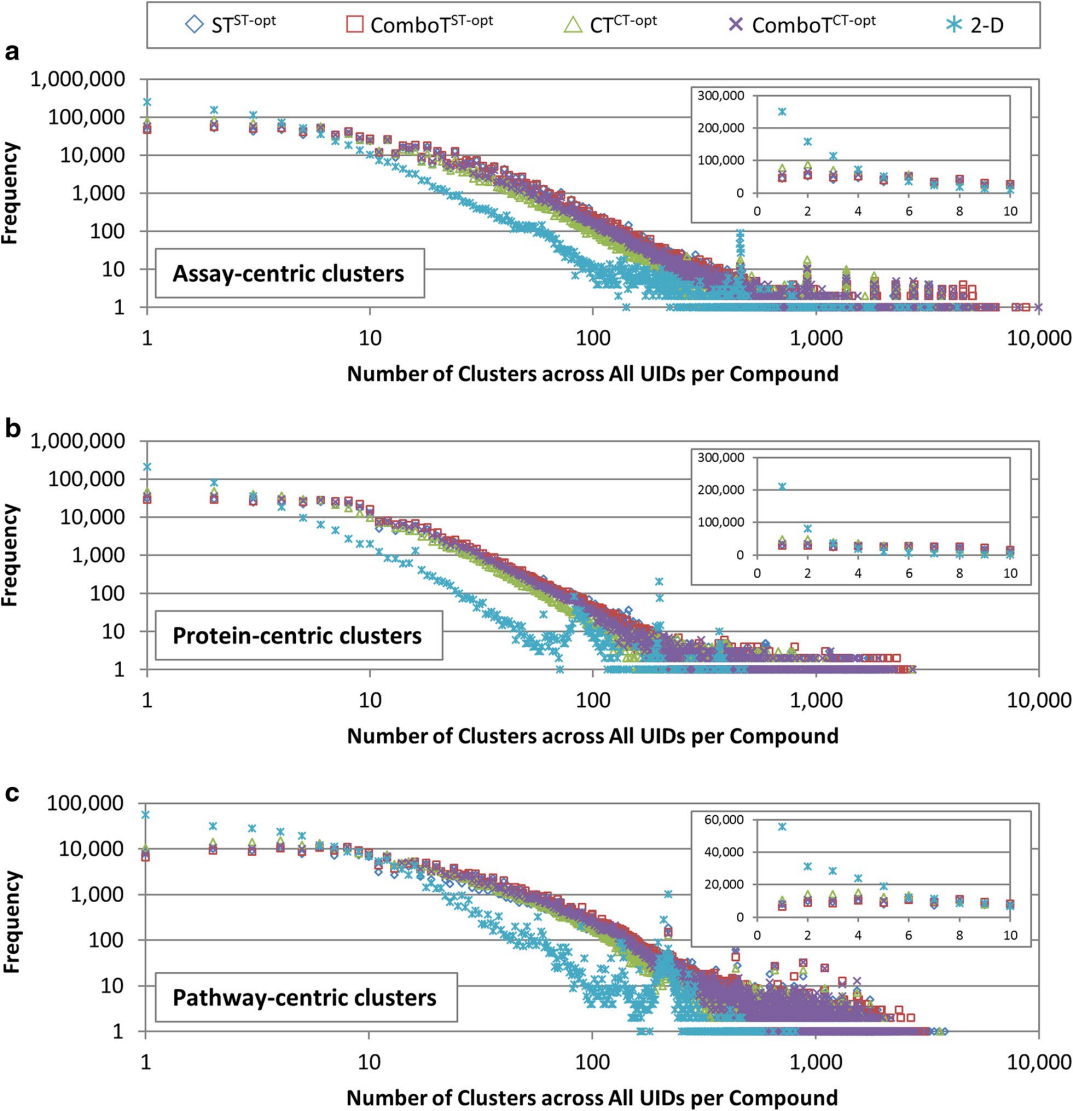
Distribution of the number of clusters across all UIDs per compound. The UID indicates AID, GI, and BSID for assay-centric (*panel* **a**), protein-centric (*panel* **b**), and pathway-centric clusters (*panel* **c**), respectively.

Figure 4 illustrates the distribution of the number of clusters per compound. Whereas the 2-D clusters were constructed through a “direct” clustering of the compounds being considered, the 3-D cluster construction involved an “indirect” clustering of the compounds, meaning that their multiple conformers were clustered first, then the conformer identifiers were converted to their corresponding compound identifiers (i.e., CIDs). For a given UID, as a result, a compound can occur in multiple 3-D clusters via its different conformers, whereas it can occur in only one 2-D cluster, as reflected in Figure 4. Many compounds occur only in one 2-D cluster across all UIDs considered for each biological similarity context. This explains why the average number of 3-D clusters per compound is much greater than the number of 2-D clusters per compound, as listed in Table 3.

### Overlap between different cluster types

One interesting question one may ask is “how similar (or different) are clusters from the five different similarity measures in the aggregate?” However, this is not an easy question to answer, considering that clustering of more than 800 thousand compounds resulted in a total of 18 million clusters. As a further complicating factor, each compound occurs in at most one 2-D cluster but can be part of any number of 3-D clusters for a given UID. In the present paper, overall similarity between the clusters from different similarity measures was estimated by the percentage of “overlapping” compounds occurring in clusters of two similarity measures relative to the total number of compounds occurring in clusters from a similarity measure, computed as

$$O(i,j) = \frac{{N_{cmpd} (i,j)}}{{N_{cmpd} (i)}} \times 100\%$$

where $N_{cmpd} (i)$ is the number of compounds occurring in clusters from similarity measure *i* for a given UID, and $N_{cmpd} (i,j)$ is the number of those occurring in clusters from both similarity measures *i* and *j* for that UID. A compound does not occur in a cluster if it was considered to be a singleton during the clustering procedure. Therefore, $O(i,j)$ quantifies the similarity in clustering behavior of both similarity measures *i* and *j* for a given UID. The cluster overlap is not necessarily symmetrical.

**Figure 5:**
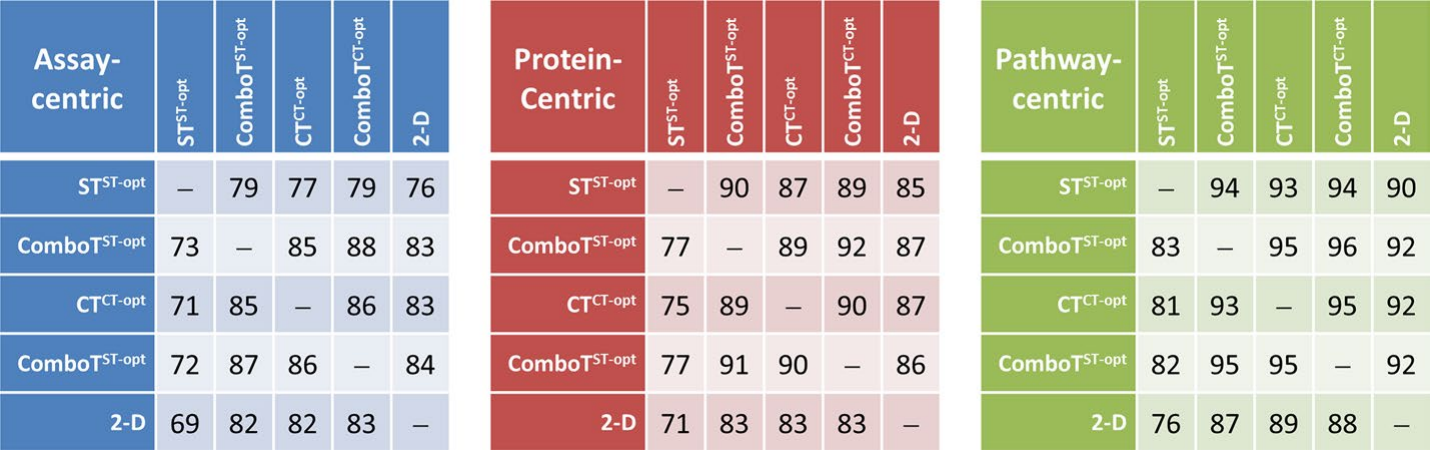
Cluster overlap between similarity measures. The overlap between clusters from five different similarity measures is quantified with the average $O(i,j)$ values, where *i* and *j* are indices for rows and columns, respectively (*see text* for the definition).

Figure 5 shows the average $O(i,j)$ values over all AIDs, GIs, and BSIDs. Among the four different 3-D cluster types, the *ST*^*ST*-*opt*^ clusters showed the least overlapping compounds with the other three 3-D clusters. For example, for the assay-centric clusters, the average values for *O*(*ST*^*ST*-*opt*^, *j*) and *O*(*i*, *ST*^*ST*-*opt*^) between *ST*^*ST*-*opt*^ and the other three 3-D clusters were 71–79%, whereas the average $O(i,j)$ values between the other three 3-D cluster types were 85% or greater. Interestingly, the *ST*^*ST*-*opt*^ clusters also showed the least overlaps with the 2-D Tanimoto similarity, with *O*(*ST*^*ST*-*opt*^, *2*-*D*) and *O*(*2*-*D*, *ST*^*ST*-*opt*^) values of 76 and 69%, respectively, which are lower than any other *O*(*i*, *2*-*D*) and *O*(*2*-*D*, *j*) values between 2-D similarity measures and the others. This may be because, among the four 3-D similarity measures considered, *ST*^*ST*-*opt*^ is the only one that does not take feature (or functional group) similarity into account. It seems that the other three 3-D similarity measures, to some extent, can take structural information into account that is encoded in molecular fingerprints by using feature atoms that represent six functional group types. However, the *ST*^*ST*-*opt*^ similarity uses steric shape of the molecule only, and this may be the reason why it produced clusters that least overlapped with those from other similarity methods used.

### SAR clusters with high-value compounds (HVCs)

By design, compounds grouped into the same PubChem SAR cluster are guaranteed to be structurally similar (in terms of one of the five similarity measures) and to have a similar bioactivity (in terms of one of the three bioactivity similarity contexts). However, what more can we say about these clusters? What is known about the compounds contained in the clusters? Given that the compounds are structurally similar and have similar bioactivity for the UID, knowing what else is known may be very helpful to characterize the meaning of the cluster. This thought led to the notion of high-value compounds (HVCs), which may provide some hints as to the nature of the cluster as defined by what is known about the compounds contained in the same cluster. An HVC was defined as a molecule whose corresponding PubChem Compound record satisfied any of the following three conditions:

1. it had a high potency, with its IC_50_ or EC_50_ value smaller than 10 μM in any bioassay archived in PubChem,
2. it had a Medical Subject Headings (MeSH) annotation [40], or
3. it had a MeSH “Pharmacological Action” annotation.

MeSH [40] is the National Library of Medicine’s controlled vocabulary thesaurus, consisting of a set of commonly used terms in the fields of health and biomedical sciences as well as medicine. The existence of a MeSH annotation to a PubChem Compound record may be an indication of a meaningful bioactivity of the molecule, evidenced by publications archived in PubMed. However, some MeSH annotations are too general (such as solvents, carcinogens, inhibitors, and so on) to describe a specific biological function of the molecule. For this reason, molecules with MeSH “Pharmacological Action” annotations were also separately included in the definition of the “high-value compounds” because these annotations indicate that a specific biological role is known. As a result, the HVCs with the “Pharmacological Action” annotation are a subset of those with the “MeSH” annotation.

**Figure 6:**
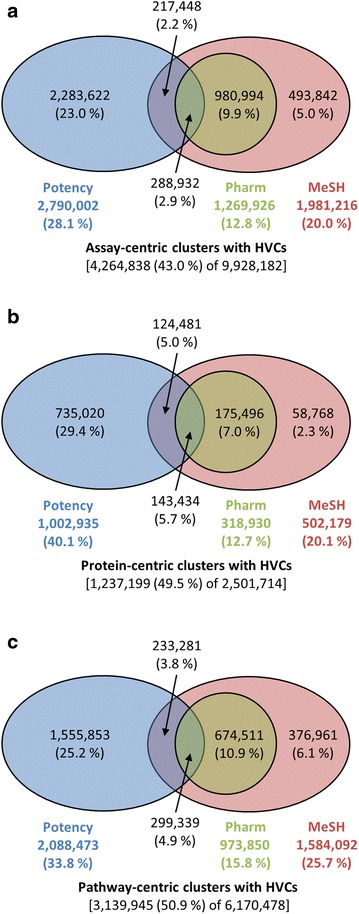
The number of the PubChem SAR clusters with high-value compounds (HVCs). The HVCs have high potencies (*blue*), MeSH annotations (*red*), or “Pharmacological Action” annotations (*green*).* Panels* **a**, **b**, and **c** are for assay-, protein-, and pathway-centric clusters. *Numbers in parentheses* indicate the percentages relative to the respective total cluster counts.

**Figure 7:**
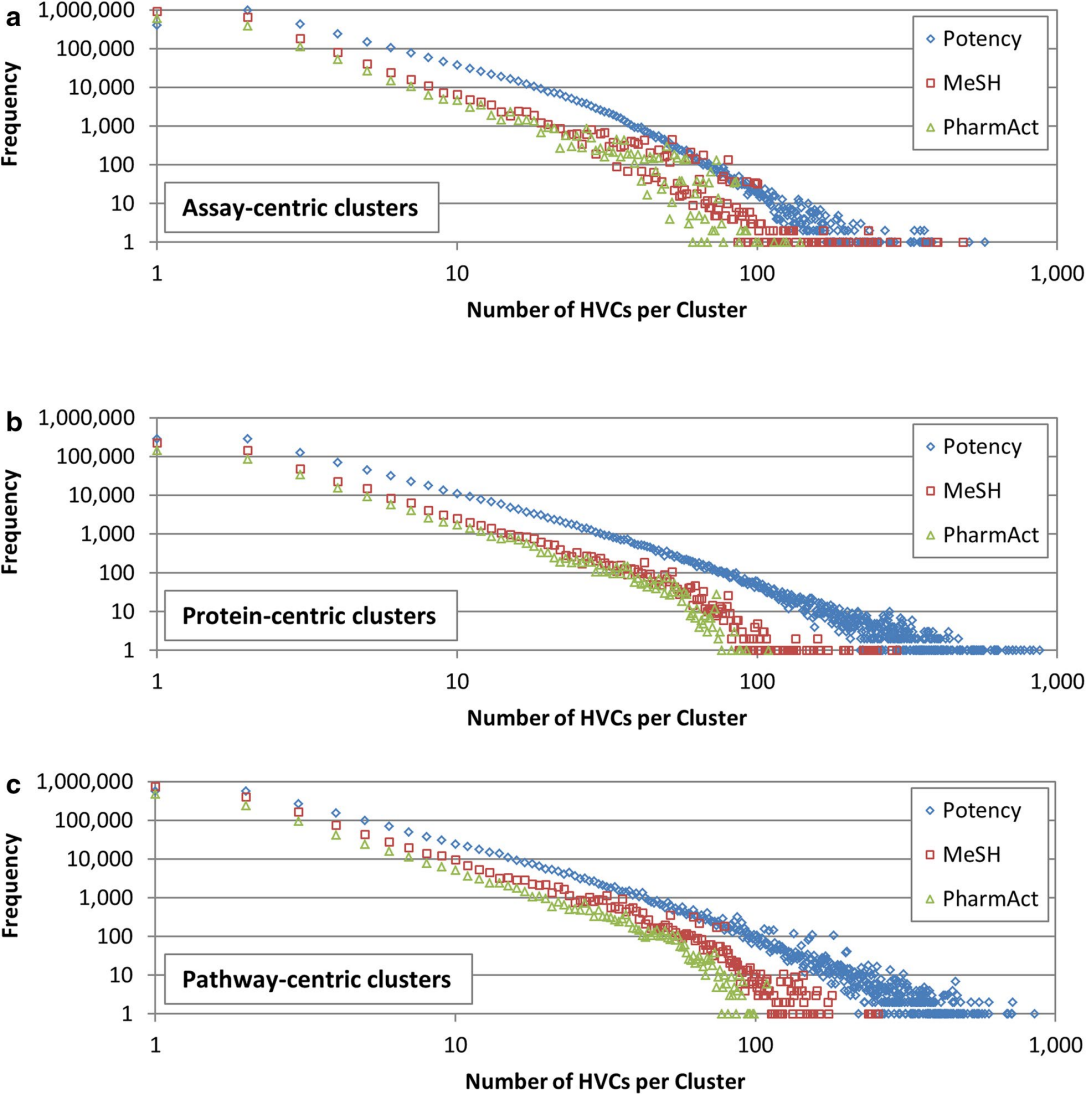
Distribution of the number of high-value compounds (HVCs) per cluster.* Panels* **a**, **b**, and **c** are for the assay-, target-, and pathway-centric clusters.

Figure 6 shows the number of clusters with HVCs for the assay-, protein-, and pathway-centric clusters. Among the 9.9 million assay-centric clusters, 43.0% (4.3 million) of them contained HVCs. The fraction of clusters containing HVCs in the protein- and pathway-centric clusters were 49.5% (1.2 million of 2.5 million clusters) and 50.9% (3.1 million of 6.2 million clusters), respectively. The clusters that have high-potency HVCs (with IC_50_ or EC_50_ values smaller than 10 μM) correspond to 28.1, 40.1, and 33.8% of the total for the assay-, protein-, and pathway-centric clusters, respectively. The clusters that have MeSH-annotation HVCs were 20.0, 20.1 and 25.7% of the total for assay-, protein-, and pathway-centric clusters, respectively. Figure 7 depicts the distribution of the number of HVCs per cluster, and the summary statistics are listed in Table 4. Some clusters have as many as hundreds of HVCs, but most clusters have only a few HVCs. On average, for example, the assay-centric clusters have 1.3 HVCs with high potency, 0.5 HVCs with MeSH, and 0.3 HVCs with Pharmacological Action annotation.

**Table 4:** Summary statistics of high-value compound (HVC) contents per cluster

	The number of HVCs			
	Potency	MeSH	Pharm	Any
Assay-based clusters				
$\bar{x}$	1.3	0.5	0.3	1.7
*s*	3.9	1.9	1.5	4.2
Minimum	0	0	0	0
Maximum	575	485	139	575
Target-based clusters				
$\bar{x}$	2.0	0.6	0.4	2.4
*s*	8.0	2.4	1.9	8.5
Minimum	0	0	0	0
Maximum	874	291	109	895
Pathway-based clusters				
$\bar{x}$	1.7	0.7	0.4	2.4
*s*	7.1	3.0	1.9	7.8
Minimum	0	0	0	0
Maximum	852	256	109	886

Symbols $\bar{x}$ and *s* indicate the average and standard deviation, respectively, of the number of HVCs per cluster.

The cut-off value of 10 μM for high-potency HVC is an arbitrary choice. Among the 4.6 million biological activities associated with the compounds considered in this study, 2.0 million (44%) indicate a biological response less than 10 μM. When these are collapsed at the compound level, 80% of the compounds are potent at less than 10 μM in at least one assay. The percentages suggest that it should be relatively common to find clusters with high-potency HVCs. In some ways, this is a fundamental point. Finding a group of chemicals that are both chemically similar and potent is the basis for determining an interesting structure–activity-relationship. The high-potency HVC may help to indicate cases of polypharmacology (e.g., where a compound has potent biological activity for another target, suggesting that the chemically similar cluster members may have similar potency this other target) that may otherwise be missed. This can make the high-potency HVC a very useful annotation for clusters.

### Examples

We have selected the following three examples that may help demonstrate the nature of the PubChem SAR clusters:

1. assay-centric clusters for AID 47904,
2. protein-centric clusters for GI 29337198, and
3. pathway-centric clusters for BSID 545294.

The PubChem SAR clusters for these UIDs are provided in Additional files 1, 2, and 3. In the examples below, some of the clusters are visualized as a compound–compound network, or conformer–conformer network, as described in the “Methods” section.

#### Carbonic anhydrase inhibitors (AID 47904)

The first example is the clusters for AID 47904, which is a literature-extracted assay that targets human carbonic anhydrase (CA) isozyme II [41]. CAs, which catalyze the interconversion between carbon dioxide and the bicarbonate ion, are involved in many important physiological processes, including respiration and transport of CO_2_/bicarbonate, pH and CO_2_ homeostasis, electrolyte secretion in a variety of tissues and organs, and biosynthetic reactions (such as gluconeogenesis, lipogenesis, and ureagenesis) [41, 42]. Therefore, CAs are considered as important therapeutic targets for many diseases, and some CA inhibitors are in clinical use mainly as diuretics and antiglaucoma agents, but also as therapeutic agents for other diseases [41, 42].

**Figure 8:**
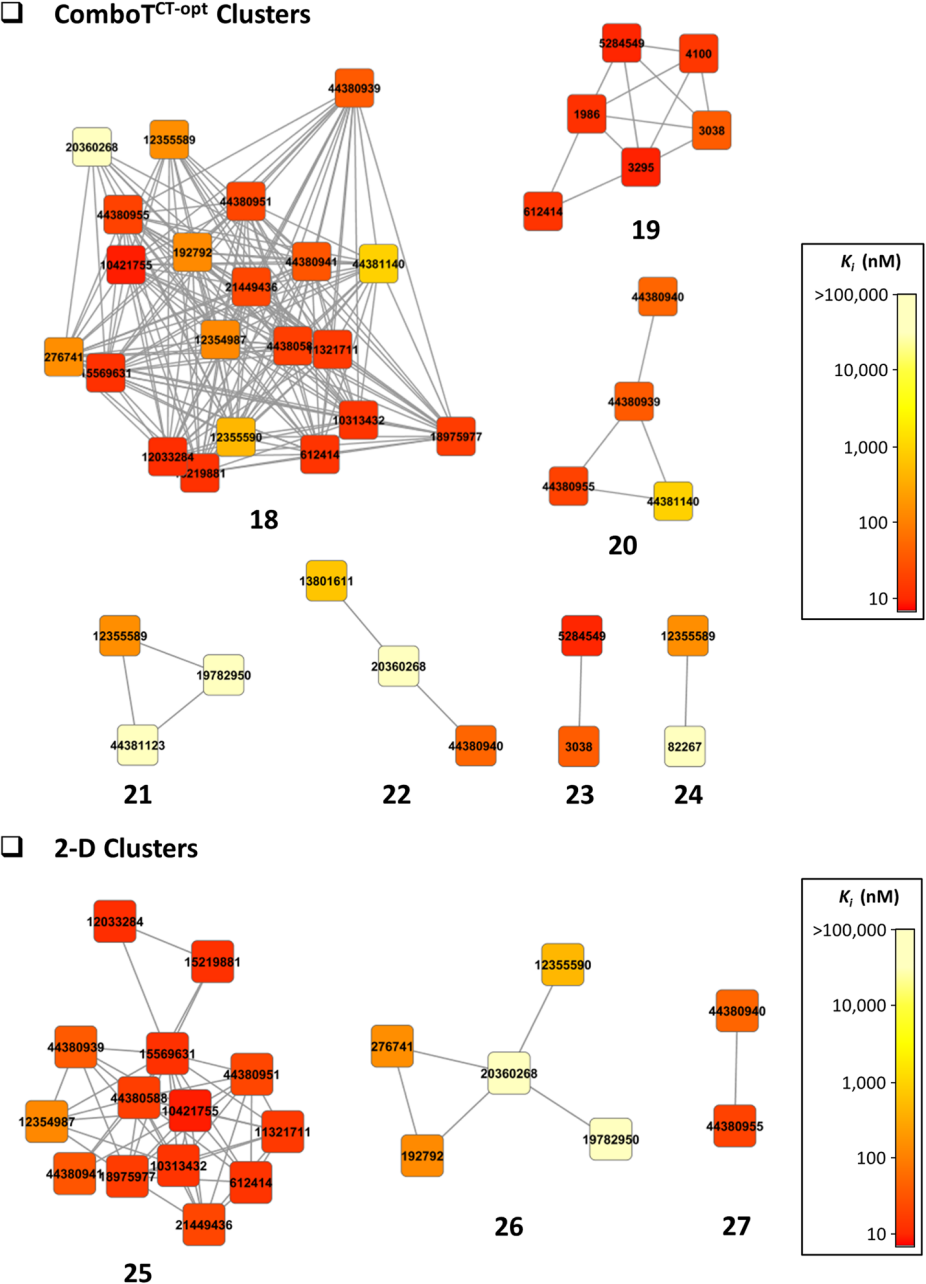
*ComboT* ^*CT*-*opt*^ and 2-D clusters for AID 47904. *Each node* represents a non-inactive compound and the edge between two nodes *within a cluster* indicates that the distance between the two CIDs is closer than the *d* ^*thresh*^ value used for clustering. The *node color* represents the value of the inhibition constant (*K*_*i*_) for the compound against human carbonic anhydrase (CA) isozyme II. All singletons are removed.

In AID 47904, sulfamide (H_2_NSO_2_NH_2_; CID 82267) and its 25 derivatives, as well as six CA inhibitors already in clinical use, were tested against human CA isozyme II. The PubChem SAR clusters (Clusters 1–27) for these 32 compounds are given as Additional file 1. The corresponding *ComboT*^*CT*-*opt*^ clusters and 2-D clusters are visualized in Figure 8, in which each node represents a compound and the edge between two nodes indicates that the distance between the two corresponding CIDs is closer than the *d*^*thresh*^ value used for clustering. When two nodes are in different clusters, no edge is added between them. However, even in this case, the two nodes may still be closer than the *d*^*thresh*^ value, which is an inevitable consequence of the clustering algorithm employed.

**Figure 9:**
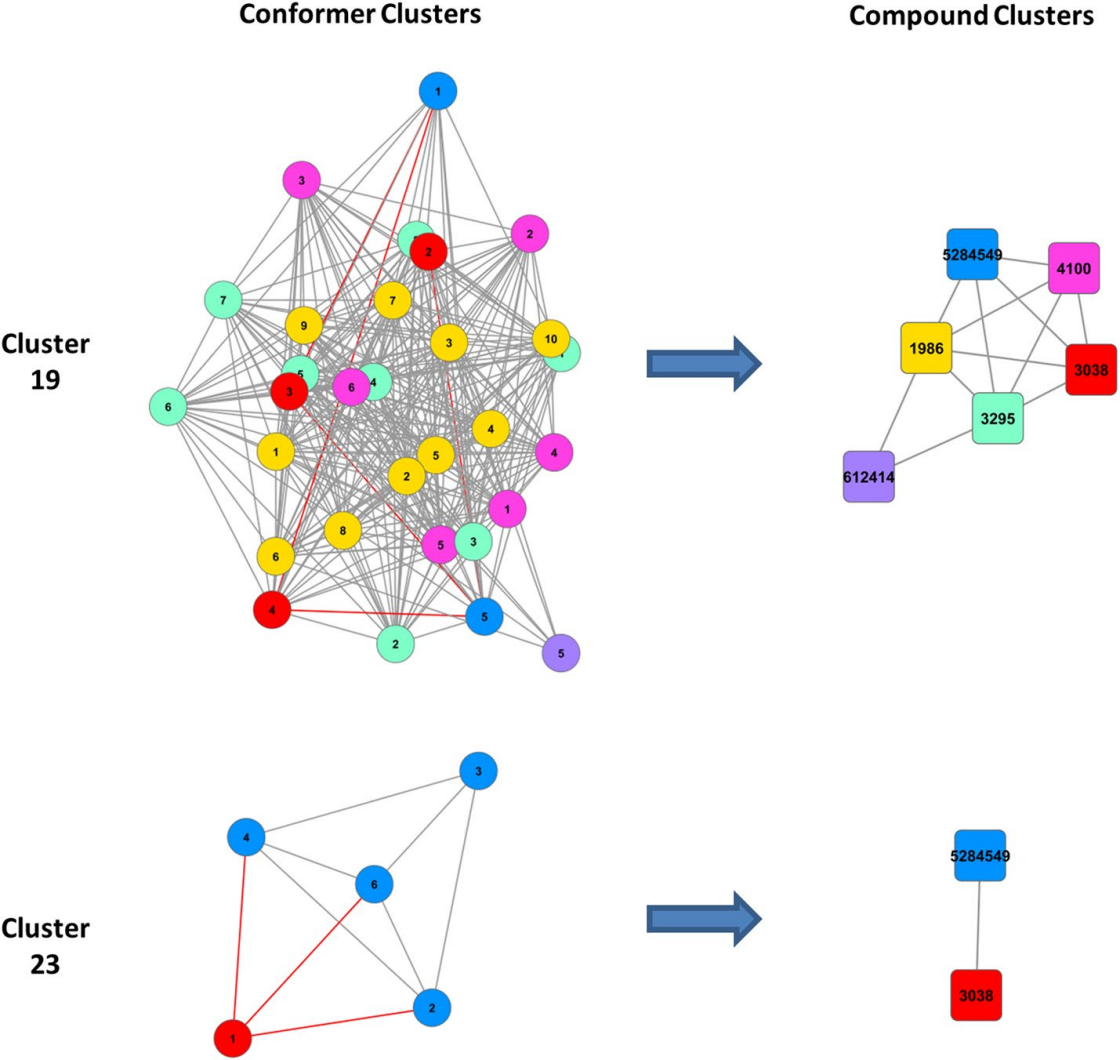
Collapse of conformer clusters into compound clusters. A compound is represented with a *square node* and its conformer is represented with a *round node* of the same color. An *edge* between two conformer nodes indicates that the distance between them is below the *d* ^*thresh*^ value used for clustering, and the *edge* between two compound nodes indicates that at least one conformer pair arising from the two compounds is below the *d* ^*thresh*^ value. PubChem 3-D SAR clustering algorithm is initially applied to conformers of non-inactive compounds, resulting in conformer clusters (in the *left panel*). Compound clusters are constructed by replacing the conformers with the respective compounds (in the *right panel*). As a result, a compound can occur in multiple compound clusters (via its different conformers).

A noticeable observation in Figure 8 is that the total number of nodes for the *ComboT*^*CT*-*opt*^ clusters is 41, which is greater than the number of non-inactive compounds used in the SAR clustering, suggesting that some of the compounds occur more than once. For example, CIDs 3038 and 5284549 occur in both Clusters 19 and 23, making Cluster 23 appear to be a subset of Cluster 19. However, this is not true at the conformer level because the conformers involved in the two clusters are not identical. Note that it is not compounds but their conformers that were clustered during the 3-D SAR clustering. As illustrated in Figure 9, a single compound can occur in different 3-D clusters via different conformers because multiple conformers per compound were used for 3-D clustering. In contrast, a compound can occur only once in 2-D clusters. This explains why there are much more 3-D clusters than 2-D clusters (as observed in Figure 1). In essence, by using up to ten conformers for each compound, the 3-D clustering considers ten times more objects than the 2-D clustering does, resulting in the increased count of 3-D clusters over 2-D clusters.

When two compounds are grouped into a common 3-D cluster via their conformers, two compounds can adopt similar 3-D shapes and, potentially, similar protein-binding features. When the two compounds occur together in multiple 3-D clusters, it indicates that the compounds share a variety of 3-D shapes. However, it should be noted that the underlying conformers in these common 3-D clusters are not necessarily the bioactive conformers. In fact, the PubChem 3-D conformer models are designed to ensure that 90% of the conformer models have at least one bioactive conformer whose root-mean-square distance (RMSD) from the experimentally determined conformation is closer than an empirically determined upper limit [43]. Not knowing which 3-D shape of a molecule is important for binding is an inherent limitation of all 3-D similarity approaches that require 3-D conformer models. Indeed, with PubChem SAR clusters there may be multiple “hypotheses” as to how a group of molecules may bind.

It is also noticeable for the example in Figure 8 that, for both the *ComboT*^*CT*-*opt*^ and 2-D, highly potent compounds tend to be clustered together. Similarly, less potent compounds are grouped together. As shown in Additional file 4: Figure S1, the compounds in Clusters 25 and 27 have aromatic rings, whereas Cluster 26 contains aliphatic sulfamides. The dendrogram in Additional file 4: Figure S1 was generated using the PubChem Structure Clustering service [44]. This service uses a single-linkage hierarchical clustering algorithm [45], which is not the same as the Taylor–Butina algorithm [36, 37] used in this study. Therefore, the clusters from the PubChem SAR clustering are not necessarily the same as those from PubChem Structure Clustering tool [44]. However, they should be closely related.

#### Agonists of aryl hydrocarbon receptor (GI 29337198)

The aryl hydrocarbon receptor (AhR, GI 29337198) [46–51] is a ligand-activated transcription factor involved in the regulation of the biological response to aromatic hydrocarbons. In the absence of agonists, it exists in the cytosol as an inactive complex with chaperone Hsp90 and co-chaperones *p*23 and ARA9. Upon agonist binding at the Per-AhR/Arnt-Sim (PAS) domain of the AhR, its association with the chaperones is altered through a conformational change, leading to translocation of the AhR from the cytoplasm to the nucleus, where it regulates gene expressions involved in detoxification and metabolism of various compounds. The acute toxicity of many environmental pollutants including halogenated dioxins such as 2,3,7,8-tetrachlorodibenzo-*p*-dioxin (TCDD; CID 15625) arises from their interactions with the AhR. In addition, because it is also involved in the regulation of cell proliferation and differentiation [50, 51], interest in AhR biology has grown, beyond a toxicological perspective, to its role in normal physiology and development of mammalian organisms [46].

In the PubChem BioAssay database, there are 13 bioassays whose target is the AhR, as listed in Table 5. These assays, deposited by ChEMBL [8], are extracted from three different scientific articles [47–49] describing studies which tested different chemical series for their ability to activate the AhR transcription using different experimental techniques and conditions. A total of 43 compounds that were tested non-inactive in at least one of the 13 bioassays are presented in Additional file 4: Figure S2, grouped according to the original publications from which the 13 assays were extracted. These compounds include 30 aurones (from PMID 20392544), 6 flavones (from PMID 19719119), and 4 imidazo[1,5-*a*]quinoxalines (from PMID 2198547), as well as three other compounds that were tested for comparison purposes [i.e., CIDs 15625 (TCDD), 2361, and 6476401]. CID 15625 was tested both in PMIDs 19719119 and 21958547.

**Table 5:** Comparison of assays targeting aryl hydrocarbon receptor (AhR, GI 29337198)

AID	Assay methods	Ligand concentration (μM)	Activity measure	Number of compounds				
				Tested	Active	Inactive	Unspecified	Non-inactive
PMID 19719119								
431863	LRGA		Fold change	7	0	0	7	7
431864	LRGA^a^	0.001	NA	1	0	1	0	0
431865	LRGA^a^	0.0003	NA	1	0	1	0	0
431866	LRGA^a^	0.0001	NA	1	0	1	0	0
431867	LRGA^a^	20	NA	1	0	1	0	0
431868	WBA	20	NA	2	2	0	0	2
431869	WBA	20	NA	2	2	0	0	2
431870	MA	20	NA	2	2	0	0	2
PMID 20392544								
490160	EROD	10	Fold change	15	0	0	15	15
490161	EROD	25	Fold change	10	0	0	10	10
490162	EROD	5	Fold change	4	0	0	4	4
490163	EROD	1	Fold change	3	0	0	3	3
PMID 21958547								
631103	CALUX	?	EC_50_	5	1	0	4	4

*LRGA* AhRE/XRE-luciferase reporter gene assay, *EROD* ethoxyresofurin-*O*-deethylase assay, *CALUX* CALUX transactivational assay, *WBA* western blot analysis, *MA* microscopic analysis with DAPI staining.

^a^In the presence of 6-hydroxy-7-methoxyflavone, which is an AhR antagonist.

**Figure 10:**
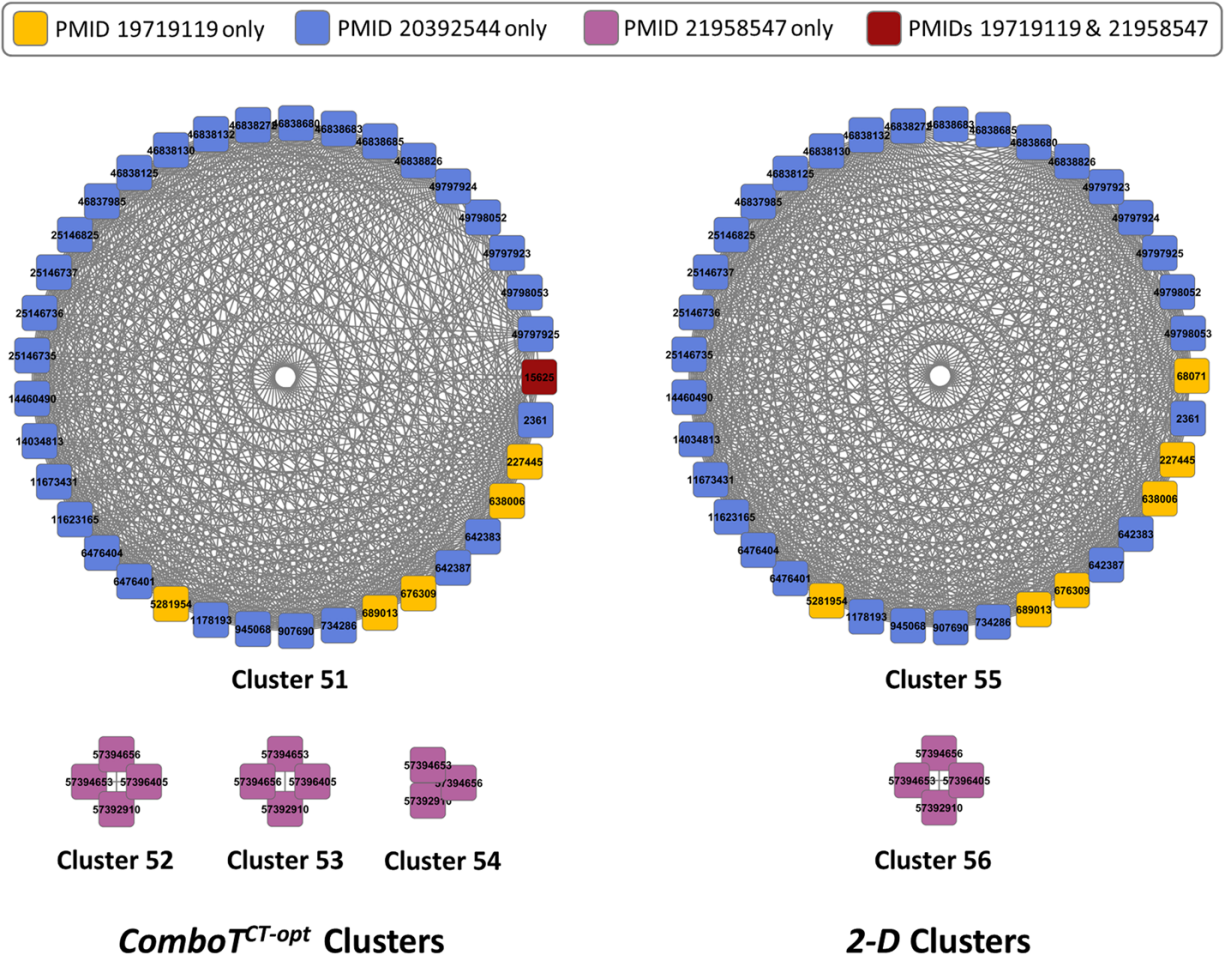
*ComboT* ^*CT*-*opt*^ and 2-D clusters for aryl hydrocarbon receptor (AhR; GI 29337198). CID 15625 (2,3,7,8-Tetrachlorodibenzo-*p*-dioxin, also known as TCDD) is tested in two different publications. The *numbers in the squares* correspond to the CIDs. The *colors of the squares* indicate the publications where data were obtained.

PubChem SAR clusters arising from these 43 compounds and a summary of their sizes are provided in Additional file 2, and the *ComboT*^*CT*-*opt*^ clusters and 2-D clusters are compared in Figure 10 for illustration purposes. The most noticeable aspect is that the flavones/isoflavones and aurones are grouped into the same cluster, indicating that there may be a structural basis for the similarity in biological activity against AhR between the two groups of chemicals, although they were tested in different published research studies using different experimental methods. It is also noteworthy that while TCDD (CID 15625) is grouped into the same 3-D cluster as flavones/isoflavones and aurones, while it is excluded as a singleton after the 2-D clustering. This illustrates how 3-D clustering can complement 2-D clustering.

This example demonstrates that the protein-centric SAR clusters provide a glimpse at the structural basis of bioactivity similarity between compounds tested in different bioassays that target a common protein. It also shows that the SAR clusters help to join data from multiple publications aiding in their trans-publication data interpretation.

#### Modulation of visual cycle I

The last example is the pathway-centric clusters for the visual cycle 1 (BSID 545294), the process of recycling all-*trans* retinal, released from the bleached pigment (such as rhodopsin in rods and cone pigment in cones) to 11-*cis*-retinal, required for pigment regeneration [52]. This record in the BioSystems database is derived from the BioCyc database collection [53] (Record PWY-6861 for human). Proteins in this pathway are targeted by 13 assays in PubChem, extracted from four scientific papers (Table 6) [54–57]. The compounds contained in these assays were tested against three different proteins involved in the visual cycle: rhodopsin (GI 129204) [54, 55], retinol-binding protein 4 (GI 62298174; RBP4) [56], and retinol dehydrogenase 9 (GI 74752227; RDH9) [57]. RDH9 is also called 3α-hydroxysteroid dehydrogenase or dehydrogenase/reductase SDR family member 9 (DHRS9), the latter of which is named after the gene that encodes the protein.

**Table 6:** Thirteen assays stored in PubChem that target the visual cycle 1 (BSID 545294)

AID	Target	Number of compounds					
		Tested	Active	Inactive	Inconclusive	Unspecified	Non-inactive
PMID 17346963							
295044	RDH9 (GI 74752227)^a^	3	1	0	0	2	3
PMID 18707087							
365154	Rhodopsin (GI 129204)	2	1	0	0	1	2
365155	Rhodopsin (GI 129204)	2	1	0	0	1	2
365156	Rhodopsin (GI 129204)	6	6	0	0	0	6
PMID 21309593							
591677	Rhodopsin (GI 129204)	14	0	0	8	6	14
PMID 21591606							
606062	RBP4 (GI 62298174)^b^	8	8	0	0	0	8
606063	RBP4 (GI 62298174)	21	0	21	0	0	0
606064	RBP4 (GI 62298174)	47	46	0	0	1	47
606065	RBP4 (GI 62298174)	1	0	0	0	1	1
606066	RBP4 (GI 62298174)	7	7	0	0	0	7
606161	RBP4 (GI 62298174)	7	7	0	0	0	7
606162	RBP4 (GI 62298174)	2	1	1	0	0	1
606163	RBP4 (GI 62298174)	7	6	1	0	0	6

^a^Retinol dehydrogenase 9. Also called 3α-hydroxysteroid dehydrogenase, or dehydrogenase/reductase SDR family member 9 (DHRS9).

^b^Retinol-binding protein 4.

**Figure 11:**
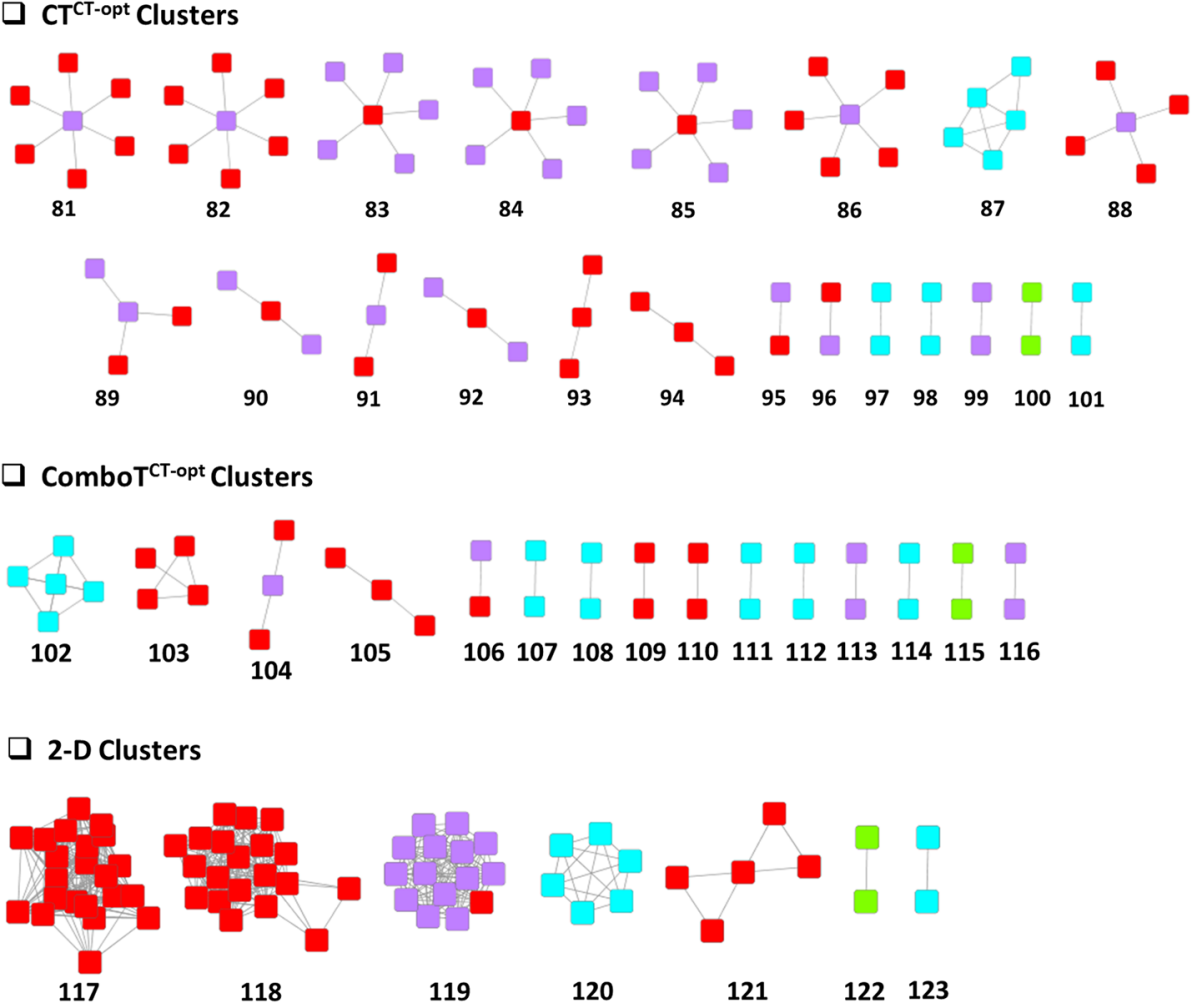
*CT* ^*CT*-*opt*^, *ComboT* ^*CT*-*opt*^, and 2-D clusters for BSID 545294. The nodes are noninactive compounds in assays involved in BSID545294. The *node colors* represent the original literature from which the biological activities of the compounds were extracted (*green* for PMID 17346963, *cyan* for PMID 18707087, *purple* for PMID 21309593, and *red* for PMID 21591606). The *node labels* are omitted for brevity, but information on cluster members can be found in Additional file 3.

The PubChem SAR clusters for BSID 545294 are provided in Additional file 3. The corresponding *CT*^*CT*-*opt*^, *ComboT*^*CT*-*opt*^, and 2-D clusters are displayed in Figure 11. For comparison purposes, the 2-D dendrogram for the 72 compounds contained in the 2-D clusters is displayed in Additional file 4: Figure S3. Most noticeable is that compounds from the same publication tend to be clustered together, except for those from PMIDs 21309593 and 21591606. Although the compounds from these two publications target different proteins in the visual cycle (rhodopsin for PMID 21309593 and RBP4 for PMID 21591606), the natural ligands of the two proteins (i.e., 11-*cis*-retinal for rhodopsin and all-*trans*-retinol for RBP4) are structurally very similar to each other: they differ by the configuration of one of their stereocenters (*trans*- vs. *cis*-configurations) and the functional group at the end of their carbon chain (hydroxyl vs. aldehyde groups). Structural similarity to these natural ligands was the basis for selection of the compound sets in the two publications.

Another important observation from Figure 11 and Additional file 4: Figure S3 is that, although the compounds from PMIDs 1870708 and 21309503 target rhodopsin, they can be classified into two groups. It is because they are believed to target different binding pockets of rhodopsin. While those from PMID 21309593 target its chromophore region where 11-*cis*-retinal covalently binds, those from PMID 1870708 target the interface in the intracellular loop where the activated rhodopsin interacts with transducin, its G-protein.

This example shows that PubChem SAR clusters help to interrelate bioactivity information across multiple publications that deal with the same biological pathway. It also illustrates that pathway-centric clusters are able to capture similarity (or dissimilarity) of chemicals that target different proteins involved in a biological pathway.

## Discussion

### Comparison of clustering with other grouping methods

Although clustering is commonly used to analyze complex data, it often adds additional complications and subjectivity. For example, the clustering algorithm employed in this study may result in two structurally similar molecules being grouped into different clusters using a given *d*^*thresh*^ clustering value. They may be grouped into the same cluster, if a different value for *d*^*thresh*^ is used. With that said, one may consider alternative grouping methods, such as grouping by scaffold or maximum common structure (MCS). These methods essentially use the 2-D representation of chemical structures. Because fingerprints used in 2-D similarity comparison also encode the 2-D structures of molecules, grouping compounds by scaffolds or MCS may be expected to give similar results to those from 2-D clustering. However, because the concept of scaffolds and MCS are essentially based on 2-D structures, but not 3-D structures, they would not necessarily be good alternatives to 3-D clustering. Considering that 3-D similarity often recognizes molecular similarity that 2-D similarity methods cannot detect, we believe that clustering using 2-D and 3-D similarity measures provides for a more complete structure–activity relationship viewpoint than grouping by scaffold or MCS.

### Future directions

The present study began as a proof-of-concept, pilot study. The aim was to explore if it was possible to provide PubChem users with quick access to pre-analyzed structure–activity relationships implicit in biological activity results. While not all compounds and bioactivities stored in PubChem today were considered in this study, the data set employed is large enough to demonstrate that the large-scale clustering of small molecules in PubChem is possible. The long-term scalability of this project is a primary concern. Pre-computation of all 3-D similarity scores and the clustering of fifteen different contexts of structural and bioactivity similarities for all PubChem data can take months on hundreds of CPU cores; however, if the biological activity data is largely static, most of this computation is a one-time cost and the results need only be stored. Improvements to the SAR cluster system are planned, including an improved user interface as well as a complete and regularly updated cluster data. A new interface is planned as a part of on-going modernization of existing PubChem interfaces to give facile access to these pre-computed clustering results.

## Conclusions

In the present study, a bioactivity-centred clustering approach was employed to group more than 800 thousand non-inactive compounds in PubChem according to their structural similarity and bioactivity similarity, resulting in a total of 18 million PubChem SAR clusters (Figure 1). Each cluster contains a group of small molecules similar to each other in both structure and bioactivity. This large-scale systematic clustering was performed under three bioactivity similarity contexts: (1) non-inactive in a given bioassay (for assay-centric clusters), (2) non-inactive against a given protein target (for protein-centric clusters), and (3) non-inactive against proteins involved in a given pathway (for pathway-centric clusters). For each context, a total of five structural similarity measures were considered: (1) $ST^{{ST{\text -}opt}}$, (2) $ComboT^{{ST{\text -}opt}}$, (3) $CT^{{CT{\text -}opt}}$, (4) $ComboT^{{CT{\text -}opt}}$, and (5) 2-D Tanimoto. The combination of the three bioactivity similarity contexts and the five structural similarity measures has led to fifteen cluster subtypes. Approximately half of the 18 million clusters contained at least one high-value compound that had high potency (with an IC_50_ or EC_50_ value of <10 μM), MeSH annotation, or Pharmacological Action annotation (Figure 6).

The summary statistics for the 2-D and 3-D SAR clusters (Table 3) indicate that the 3-D clustering resulted in more but smaller clusters than the 2-D clustering. This is a result of two important characteristics of the 3-D clustering. First, the 3-D clustering “indirectly” groups compounds, by clustering their conformers first, and then collapsing conformers to corresponding compounds. Second, the 3-D clustering uses multiple (up to ten) conformers per compound. Therefore, in the 3-D clustering, a compound may occur multiple times in different 3-D clusters (via different conformers) for a given UID. As a consequence, 3-D clustering results in more clusters than the 2-D clustering, which does not use conformers of a molecule. This interpretation is consistent with the examples provided in this study (as illustrated in Figures 8, 11).

The three examples selected in the present study illustrate some important characteristics of the PubChem SAR clusters, such as difference between the 2-D and 3-D clusterings. They show how PubChem SAR clustering helps to organize information on compounds that target a common protein but that are scattered in different assays. It was also demonstrated that compounds targeting different proteins involved in a given biological pathway can be organized according to their target proteins and binding pockets. In addition, PubChem SAR clusters can aid in the interpretation of bioactivity data scattered across multiple publications.

The SAR clusters derived from the present study are available at the PubChem SAR clusters homepage [29]. These clusters enable PubChem users to quickly navigate and narrow down the compounds of interest. Each derived SAR cluster can be a useful resource in developing a meaningful SAR or enable one to design or expand compound libraries from the cluster. It can also help to predict the potential therapeutic effect and pharmacological actions for less-known compounds from those well-known compounds (e.g., drugs) in the same SAR cluster.

## Methods

### Datasets

In the present study, the SAR clusters were constructed for three different sets (i.e., **Sets A**, **B**, and **C)** of compounds that were tested to be “non-inactive” in at least one assay archived in the PubChem BioAssay database [4] (as of September 2010). Non-inactive molecules are those which are not inactive against the assay target, including “unspecified” and “inconclusive” compounds as well as “active” molecules. The reason for considering non-inactive compounds, rather than active compounds, in the SAR clustering is that, in many assays, the unspecified and inconclusive compounds are indeed active in many assays. For example, the unspecified compounds in some assays in Table 5 do have AhR agonist activities (given in fold induction of AhR expression compared to the untreated controls), but the assay contributor did not explicitly specify whether they are active or inactive. In addition, although TCDD (CID 15625) is the strongest AhR agonist in both AIDs 631103 and 431863, it was defined as “active” only in AID 631103, but not in AID 431863. In this sense, the use of non-inactive compounds, rather than active compounds, somehow reflects the heterogeneous nature of the PubChem Bioassay data because the activity outcome of the compounds tested in PubChem bioassays is defined by the individual depositors, not by PubChem.

The three compound sets (**Sets A**, **B**, and **C**) are different from one another in the context in which their bioactivities are interpreted. As listed in Table 1, **Set A** consisted of 843,845 compounds that had a PubChem3D conformer model available *and* that were tested to be non-inactive in at least one of the 548,071 biological assays archived in the PubChem BioAssay database (as of September 2010). **Set B** consisted of 400,599 compounds with 3-D structures that were tested to be non-inactive against at least one of the 4,280 protein targets associated with the biological assays considered. **Set C** had 265,470 compounds with 3-D description that were non-inactive against proteins involved in at least one of the 4,540 biological pathways associated with the biological assay considered.

Bioactivity information of compounds tested in each of the biological assays considered was retrieved from the PubChem BioAssay database [4] and used to construct **Set A**. The construction of **Set B** requires knowledge of the protein targets of the assays considered. Note that, although multiple assays may be performed against an identical protein sequence, the assay-target association information deposited in PubChem may *not* be identical, because the target sequence can have multiple different identifiers [e.g., the same protein with different GI numbers (NCBI sequence identifier) and potentially from different organisms]. PubChem addresses this issue by assigning each assay target to a protein identity group (PIG), which is determined on the basis of the protein sequence identity. As a result, the identical protein sequence tested in different assays will belong to the same PIG (although they can still have different GI numbers). The Entrez link “pcassay_protein_target_pig” allows the user to retrieve the GI’s of all the protein sequences identical to the target protein of an assay. Information on what pathway the protein target of an assay was involved in, which was necessary to construct **Set C**, was retrieved through the Entrez link “pcassay_biosystems”. This link provides the BioSystems identifiers (BSID) [35] for the biological pathways associated with a given assay in PubChem. In conjunction with the NCBI’s FLink [58], these two Entrez links can be used to bulk download information on the protein targets and pathways associated for multiple assays.

Not all assays in PubChem have protein target information because some assays were performed against a cell line or organism, rather than a specific protein target. Similarly, some assays do not have associated pathway information from the BioSystems database [35]. As a result, the number of compounds in **Sets B** and **C** are 47% and 31% of that in **Set A** (Table 1), respectively.

### PubChem 2-D and 3-D similarity metrics

The PubChem subgraph fingerprint [9], which encodes structural information of a molecule into a binary vector of 881 substructures, is used to evaluate the 2-D similarity between two molecules, in conjunction with the Tanimoto coefficient [10–12],

1 $$Tanimoto = \frac{AB}{A + B - AB}$$

where *A* and *B* are the respective counts of fingerprint set bits in the compound pair and *AB* is the count of bits in common.

In addition to the 2-D similarity measure using the PubChem fingerprint and Tanimoto equation, PubChem uses two 3-D similarity metrics: shape-Tanimoto (ST) [19, 21, 22, 25–28] and color-Tanimoto (CT) [19, 21, 22, 26]. The ST score is a measure of shape similarity, which is defined as the following:

2 $$ST = \frac{{V_{AB} }}{{V_{AA} + V_{BB} - V_{AB} }}$$

where *V*_*AA*_ and *V*_*BB*_ are the self-overlap volumes of conformers A and B and *V*_*AB*_ is the common overlap volumes between them. The CT score, given as the following, quantifies the similarity of 3-D functional group similarity between two conformers [25, 26]:

3 $$CT = \frac{{\sum\limits_{f} {V_{AB}^{f} } }}{{\sum\limits_{f} {V_{AA}^{f} } + \sum\limits_{f} {V_{BB}^{f} } - \sum\limits_{f} {V_{AB}^{f} } }}$$

where the index “*f*” indicates any of six functional group types (i.e., hydrogen-bond donors, hydrogen-bond acceptors, cations, anions, hydrophobes, and rings), represented by fictitious “feature” or “color” atoms, $V_{AA}^{f}$ and $V_{BB}^{f}$ are the self-overlap volumes for feature atom type *f*, and $V_{AB}^{f}$ is the overlap volume of conformers A and B for feature atom type *f*. These similarity metrics can be combined to create a Combo-Tanimoto (ComboT) [21, 22, 25, 26], as specified by Eq. (4):

4 $$ComboT = ST + CT$$

Because the ST and CT scores range from 0 (for no similarity) to 1 (for identical molecules), the ComboT score may have a value from 0 to 2 (without normalization to unity).

The ST, CT, and ComboT scores between two molecules can be evaluated in two different molecular superpositions [24–26]: (1) in the ST- or shape-optimized superposition, and (2) in the CT- or feature-optimization superposition. In the shape-optimization, the superposition of two molecules is optimized to have a maximum ST score. In the feature-optimization, both color and shape of the two conformers is considered simultaneously to find the best superposition between them. For clarification, the optimization type is denoted with superscript, “ST-opt” or “CT-opt”. As a result, there are six different 3-D similarity score types used in PubChem: $ST^{{ST{\text -}opt}}$, $CT^{{ST{\text -}opt}}$, $ComboT^{{ST{\text -}opt}}$, $ST^{{CT{\text -}opt}}$, $CT^{{CT{\text -}opt}}$, and $ComboT^{{CT{\text -}opt}}$. In the present study, the SAR clusters were constructed using five different similarity measures: $ST^{{ST{\text -}opt}}$, $ComboT^{{ST{\text -}opt}}$, $CT^{{CT{\text -}opt}}$, $ComboT^{{CT{\text -}opt}}$, and 2-D Tanimoto. The SAR cluster construction was performed only for four of the six 3-D similarity measures (two measures for each optimization type), because knowledge of the ComboT score and either of the ST or CT scores is enough to get the other one [according to Eq. (4)].

### 3-D conformer models

The conformer models used for the 3-D similarity score computation were downloaded from PubChem. The PubChem conformer generation and sampling procedures, described in more detail in our previous papers [17, 21, 23, 24], ensure that 90% of the conformer models have at least one “bioactive” conformer whose (non-hydrogen atom pair-wise) RMSD from the experimentally determined conformation was closer than the upper-limit value predicted using an empirically derived equation [43]. Although each of these conformer models contains up to 500 conformers, it is not practical to consider all conformers for 3-D similarity computation. Therefore, the present study used up to ten diverse conformers per compound [21].

### 3-D similarity score pre-computation

The clustering algorithm employed in the present study requires a distance matrix, each element of which represents the distance or dissimilarity between two compounds being considered for clustering. This dissimilarity was computed by subtracting from unity the similarity score between the two compounds. The 2-D similarity scores were computed on the fly when the distance matrix was assembled. However, because 3-D similarity score calculation is much more computationally expensive, the four 3-D similarity scores (i.e., $ST^{{ST{\text -}opt}}$, $ComboT^{{ST{\text -}opt}}$, $CT^{{CT{\text -}opt}}$, and $ComboT^{{CT{\text -}opt}}$) between two compounds were “pre-computed” if both compounds were tested to be non-inactive in at least one common bioassay. All the 3-D similarity scores were saved in a data warehouse with the respective translation/rotation matrices that give the conformer superpositions at which the similarity scores were evaluated. These stored scores were retrieved from the data warehouse when the distance matrix was assembled for 3-D clustering.

### Cluster generation

The SAR clusters were constructed using the Taylor–Butina grouping algorithm [36–39] with each of the five different similarity measures: $ST^{{ST{\text -}opt}}$, $ComboT^{{ST{\text -}opt}}$, $CT^{{CT{\text -}opt}}$, $ComboT^{{CT{\text -}opt}}$, and 2-D Tanimoto. This iterative non-hierarchical clustering algorithm begins with the identification of the compound that is similar to the most compounds, given a similarity exclusion (distance) threshold. That compound is chosen as cluster representative and forms the first cluster with those compounds that are within its exclusion region determined by the distance threshold. Clustered compounds are excluded from further consideration. This process is repeated until there are no more compounds that form new compound clusters. A more detailed description of the Taylor–Butina algorithm can be found elsewhere [36–39].

The distance thresholds used for the SAR cluster construction were chosen based on the results of our recent studies on the similarity score distributions for randomly selected biologically tested compounds (Table 2) [17, 24]. The overall average ($\bar{x}$) and standard deviation (*s*) of the 3-D similarity scores between randomly selected compounds were 0.65 ± 0.10, 0.77 ± 0.13, 0.25 ± 0.07, and 0.77 ± 0.14, for $ST^{{ST{\text -}opt}}$, $ComboT^{{ST{\text -}opt}}$, $CT^{{CT{\text -}opt}}$, and $ComboT^{{CT{\text -}opt}}$, respectively, when up to ten conformers were employed for each compound. The overall average and standard deviation for the 2-D similarity scores were 0.42 ± 0.13. The distance threshold for each of the five similarity measures was selected to be the respective [1 − ($\bar{x}$ + 2 *s*)] value (after normalization to unity for *ComboT*^*ST*-*opt*^ and *ComboT*^*CT*-*opt*^). An underlying assumption for this choice is that any two compounds with a similarity score greater than the $\bar{x}$ + 2 *s* value are considered to be structurally similar to each other, which may suggest biological similarity. The distance threshold selection involved a conversion of these “similarity” thresholds into the “dissimilarity” thresholds by subtracting them from unity.

Because the 3-D similarity comparison between compounds requires conformers of the compounds, the 3-D clustering algorithm was also applied to the conformers, resulting in clusters of conformers. Then, the conformer clusters were collapsed into compound clusters, by converting conformer identifiers into corresponding compound identifiers (CIDs). That is, the 3-D SAR clusters (of compounds) were “indirectly” generated via 3-D clustering of their conformers. On the contrary, the 2-D clustering, which does not use conformers, was “directly” applied to the compounds. This difference between the 2-D and 3-D clusterings leads to a substantial difference in size and number of the resulting clusters, as shown in the “Results” section.

### Visualization of clusters

For illustration purposes, the SAR clusters were visualized as compound–compound or conformer–conformer networks, using Cytoscape [59]. Compounds are represented by square nodes, conformers by round nodes. If possible, each compound node was labelled with the CID, and each conformer node was labelled with the local conformer ID [21], which was a positive integer. When the nodes were too small to be labelled, the labels were omitted, but one can still find information on cluster members in Additional files 1, 2, and 3. An edge between two conformer nodes indicates that the distance between them was closer than the *d*^*thresh*^ value used for the SAR clustering. An edge between two compound nodes indicates that at least one conformer pair arising from the two compounds was closer than the *d*^*thresh*^ value.

## Additional files

Additional file 1: (example1_aid_47904.txt): contains the results of PubChem SAR clustering (Clusters 1–27) for AID 47904 (CA inhibitors). Additional file 2: (example2_gi_29337198.txt): contains the results of PubChem SAR clustering (Clusters 28–56) for GI 29337198 (AhR agonists). Additional file 3: (example3_bsid_545294.txt): contains the results of PubChem SAR clustering (Clusters 57–123) for BSID 545294 (visual cycle 1). Additional file 4: (supplementary_data.pdf): contains Figures S1, S2, and S3.
