# Supplementary material for: PubChem structure–activity relationship (SAR) clusters
Source: J Cheminform. 2015 Jul 7;7:33. doi: 10.1186/s13321-015-0070-x (PMC4492103; doi:10.1186/s13321-015-0070-x)
Supplement: Additional file 4: — (supplementary_data.pdf): contains Figures S1, S2, and S3. [file 13321_2015_70_MOESM4_ESM.pdf]

Supplementary Data

PubChem structure-activity relationship (SAR) clusters

Sunghwan Kim*, Lianyi Han*, Bo Yu, Volker D. Hähnke, Evan E. Bolton§ and Stephen H. Bryant

National Center for Biotechnology Information, National Library of Medicine, National Institutes of Health, Department of Health and Human Services, 8600 Rockville Pike, Bethesda, MD 20894, USA

*These authors contributed equally to this work.
§Corresponding author (bolton@ncbi.nlm.nih.gov)

Contents

Figure S1. 2-D Tanimoto Dendrogram for compounds contained in Clusters 25, 26, and 27 of AID 47904. The 21 compounds contained in the 2-D clusters for AID 47904 can be classified into two classes: aromatic sulfamides (Clusters 25 and 27) and aliphatic sulfamides (Cluster 26).

Figure S2. **Non-inactive compounds against aryl hydrocarbon receptor (AhR; GI 29337198).** These 43 compounds are tested to be non-inactive against AhR in 13 literature-extracted assays derived from three publications. CID 15625 (2,3,7,8-Tetrachlorodibenzo-*p*-dioxin, also known as TCDD) is tested in two different publications.

Figure S3. 2-D dendrogram for 72 compounds contained in 2-D clusters for BSID 545294. The compounds tested against the visual cycle can be grouped into several scaffolds, which are believed to target different proteins, or different binding pockets of the same protein.

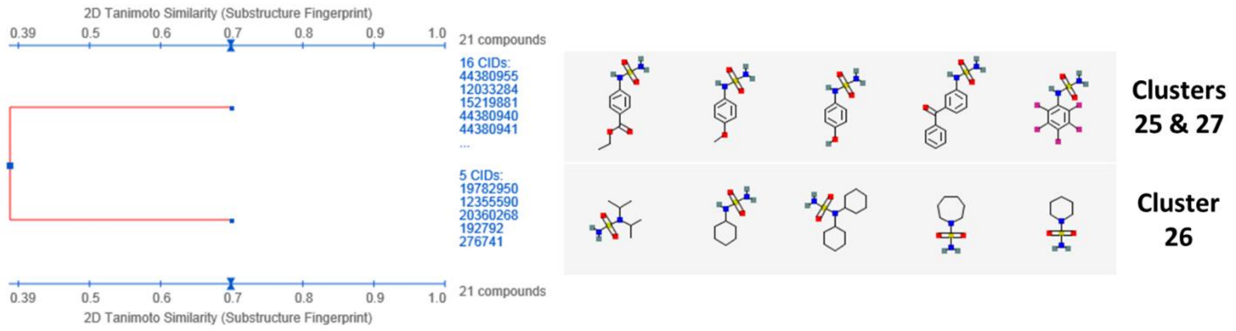

Figure S1. 2-D Tanimoto Dendrogram for compounds contained in Clusters 25, 26, and 27 of AID 47904. The 21 compounds contained in the 2-D clusters for AID 47904 can be classified into two classes: aromatic sulfamides (Clusters 25 and 27) and aliphatic sulfamides (Cluster 26).

❑ **PMID 19719119 (7 CIDs): TCDD (CID 15625) and 6 flavones/isoflavones**

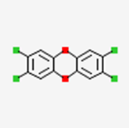
CID 15625
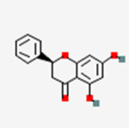
CID 68071
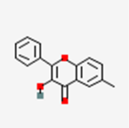
CID 227445
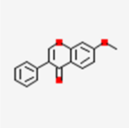
CID 638006
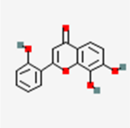
CID 676309
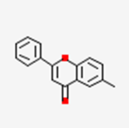
CID 689013
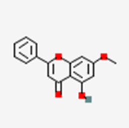
CID 5281954

❑ **PMID 20392544 (32 CIDs): β-naphthoflavone (CID 2361), sulforaphane (CID 5350), and 30 aurones**

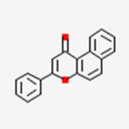
CID 2361
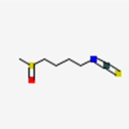
CID 5350
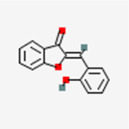
CID 642383
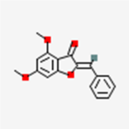
CID 642387
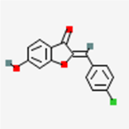
CID 734286
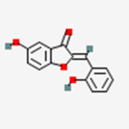
CID 907690
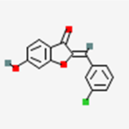
CID 945068
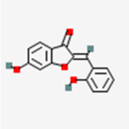
CID 1178193
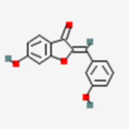
CID 6476401
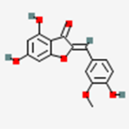
CID 6476404
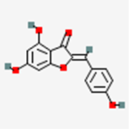
CID 11623165
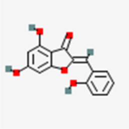
CID 11673431
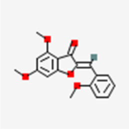
CID 14034813
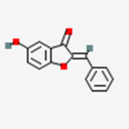
CID 14460490
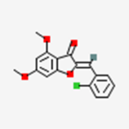
CID 25146735
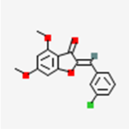
CID 25146736
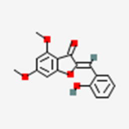
CID 25246737
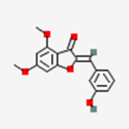
CID 25146825
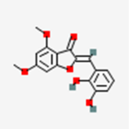
CID 46837985
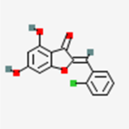
CID 46838125
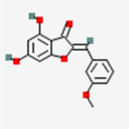
CID 46838130
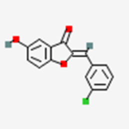
CID 46838132
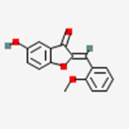
CID 46838272
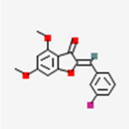
CID 46838680
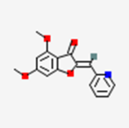
CID 46838683
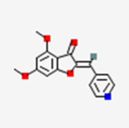
CID 46838685
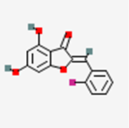
CID 46838826
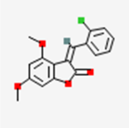
CID 49797923
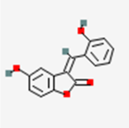
CID 49797924
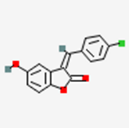
CID 49797925
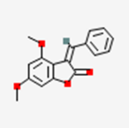
CID 49797952
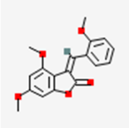
CID 49797953

❑ **PMID 21958547 (5 CIDs): TCDD (CID 15625) and 4 imidazo[1,5-a]quinoxalines**

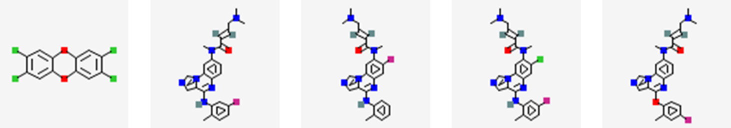
CID 15625
CID 57392910
CID 57394653
CID 57394656
CID 57396405

Figure S2. **Non-inactive compounds against aryl hydrocarbon receptor (AhR; GI 29337198).** These 43 compounds are tested to be non-inactive against AhR in 13 literature-extracted assays derived from three publications. CID 15625 (2,3,7,8-Tetrachlorodibenzo-*p*-dioxin, also known as TCDD) is tested in two different publications.

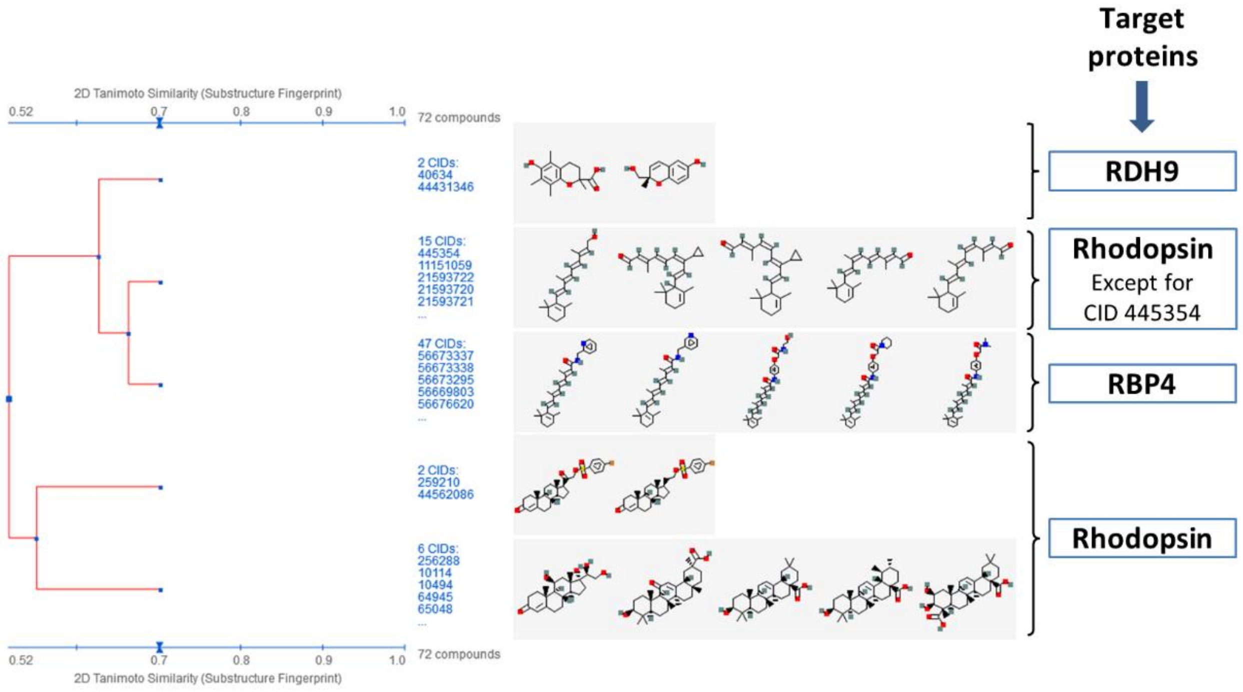

Figure S3. 2-D dendrogram for 72 compounds contained in 2-D clusters for BSID 545294. The compounds tested against the visual cycle can be grouped into several scaffolds, which are believed to target different proteins, or different binding pockets of the same protein.
